# Supplementary material for: Deciphering the spatial landscape and plasticity of immunosuppressive fibroblasts in breast cancer
Source: Nat Commun. 2024 Apr 1;15:2806. doi: 10.1038/s41467-024-47068-z (PMC10984943; doi:10.1038/s41467-024-47068-z)
Supplement: Supplementary file 1 — Supplementary Information [file 41467_2024_47068_MOESM1_ESM.pdf]

SUPPLEMENTARY INFORMATION for

Deciphering the spatial landscape and plasticity of immunosuppressive fibroblasts in breast cancer

Hugo Croizer[1,2,#], Rana Mhaidly[1,2,#], Yann Kieffer[1,2,#], Geraldine Gentric[1,2,], Lounes Djerroudi[1,2,3], Renaud Leclere[3], Floriane Pelon[1,2], Catherine Robley[1,2], Mylene Bohec[4,5], Arnaud Meng[1,2], Didier Meseure[3], Emanuela Romano[6], Sylvain Baulande[4,5], Agathe Peltier[1,2], Anne Vincent-Salomon[3] and Fatima Mechta-Grigoriou[1,2,*]

[#] These authors contributed equally

[1] Institut Curie, Stress and Cancer Laboratory, Equipe labélisée par la Ligue Nationale contre le Cancer, PSL Research University, 26, rue d'Ulm, F-75248 Paris, France

[2] Inserm, U830, 26, rue d'Ulm, Paris, F-75005, France

[3] Department of Diagnostic and Theragnostic Medicine, Institut Curie Hospital Group, 26, rue d'Ulm, F-75248 Paris, France

[4] Institut Curie, PSL Research University, ICGex Next-Generation Sequencing platform, 75005 Paris, France

[5] Institut Curie, PSL Research University, Single Cell Initiative, 75005 Paris, France

[6] Department of Medical Oncology, Center for Cancer Immunotherapy, Institut Curie, 26, rue d'Ulm, F-75248 Paris, France

* Corresponding: Fatima Mechta-Grigoriou. Phone: +33 (0)1 56 24 66 53; Fax: +33 (0)1 56 24 66 50; E-mail address: fatima.mechta-grigoriou@curie.fr

Running title: Topology and plasticity of FAP+ CAF populations

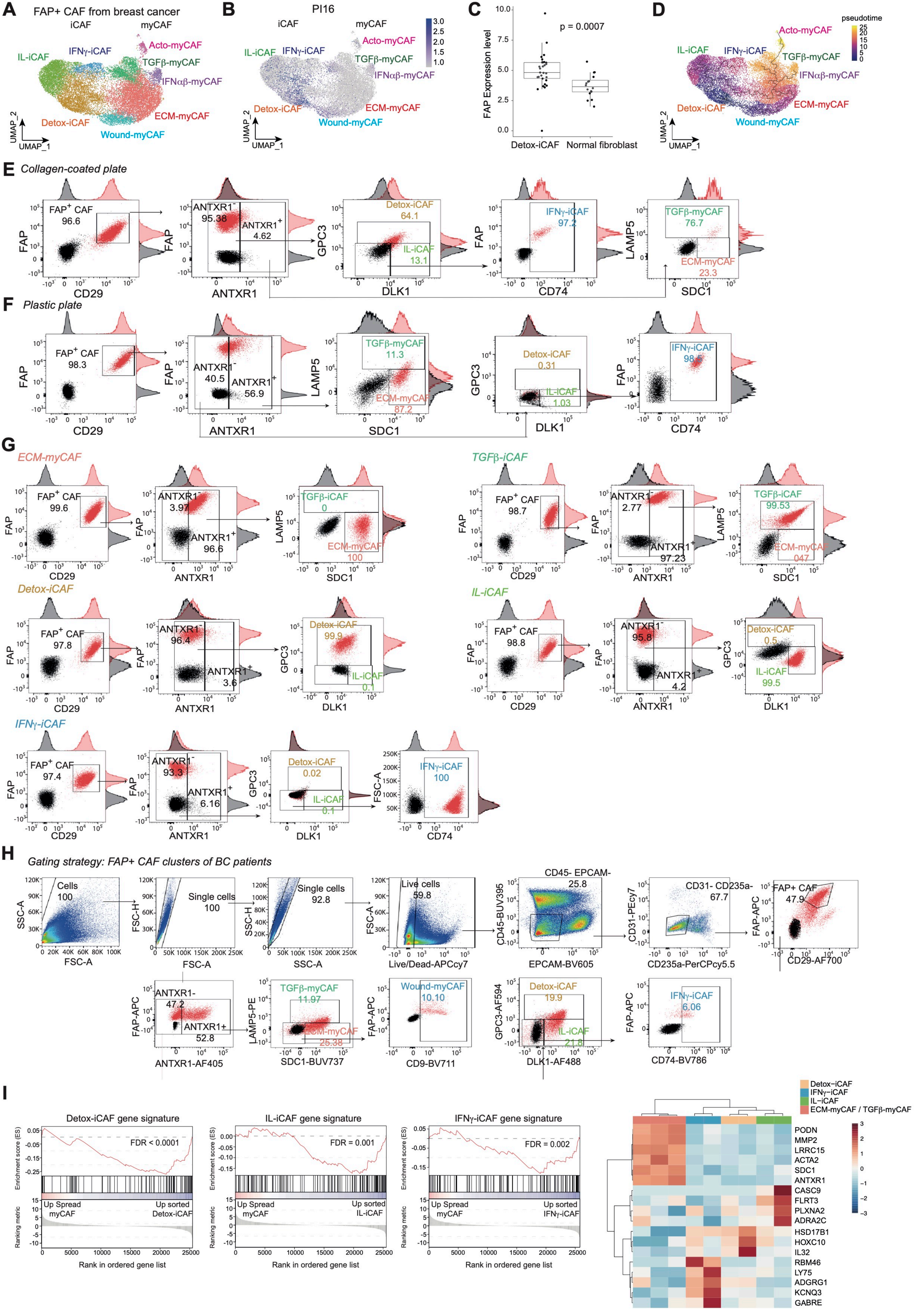
A
FAP+ CAF from breast cancer
iCAF
myCAF
IL-iCAF
IFNγ-iCAF
Acto-myCAF
TGFβ-myCAF
IFNαβ-myCAF
ECM-myCAF
Detox-iCAF
Wound-myCAF
UMAP_1
UMAP_2
B
PI16
C
FAP Expression level
p = 0.0007
Detox-iCAF
Normal fibroblast
D
pseudotime
E
Collagen-coated plate
FAP+ CAF
96.6
ANTXR1-
95.38
ANTXR1+
4.62
Detox-iCAF
64.1
IL-iCAF
13.1
IFNγ-iCAF
97.2
TGFβ-myCAF
76.7
ECM-myCAF
23.3
CD29
ANTXR1
DLK1
CD74
SDC1
FAP
GPC3
LAMP5
F
Plastic plate
FAP+ CAF
98.3
ANTXR1-
40.5
ANTXR1+
56.9
TGFβ-myCAF
11.3
ECM-myCAF
87.2
Detox-iCAF
0.31
IL-iCAF
1.03
IFNγ-iCAF
98.5
G
ECM-myCAF
FAP+ CAF
99.6
ANTXR1-
3.97
ANTXR1+
96.6
TGFβ-iCAF
0
ECM-myCAF
100
TGFβ-iCAF
FAP+ CAF
98.7
ANTXR1-
2.77
ANTXR1+
97.23
TGFβ-iCAF
99.53
ECM-myCAF
047
Detox-iCAF
FAP+ CAF
97.8
ANTXR1-
96.4
ANTXR1+
3.6
Detox-iCAF
99.9
IL-iCAF
0.1
IL-iCAF
FAP+ CAF
98.8
ANTXR1-
95.8
ANTXR1+
4.2
Detox-iCAF
0.5
IL-iCAF
99.5
IFNγ-iCAF
FAP+ CAF
97.4
ANTXR1-
93.3
ANTXR1+
6.16
Detox-iCAF
0.02
IL-iCAF
0.1
IFNγ-iCAF
100
FSC-A
H
Gating strategy: FAP+ CAF clusters of BC patients
Cells
100
Single cells
100
Single cells
92.8
Live cells
59.8
CD45- EPCAM-
25.8
CD31- CD235a-
67.7
FAP+ CAF
47.9
SSC-A
FSC-A
FSC-H
SSC-H
Live/Dead-APCcy7
CD45-BUV395
EPCAM-BV605
CD31-PEcy7
CD235a-PerCPcy5.5
FAP-APC
CD29-AF700
ANTXR1-
47.2
ANTXR1+
52.8
TGFβ-myCAF
11.97
ECM-myCAF
25.38
Wound-myCAF
10.10
Detox-iCAF
19.9
IL-iCAF
21.8
IFNγ-iCAF
6.06
ANTXR1-AF405
LAMP5-PE
SDC1-BUV737
CD9-BV711
GPC3-AF594
DLK1-AF488
CD74-BV786
I
Detox-iCAF gene signature
FDR < 0.0001
Up Spread myCAF
Up sorted Detox-iCAF
IL-iCAF gene signature
FDR = 0.001
Up sorted IL-iCAF
IFNγ-iCAF gene signature
FDR = 0.002
Up sorted IFNγ-iCAF
Enrichment score (ES)
Ranking metric
Rank in ordered gene list
Detox-iCAF
IFNγ-iCAF
IL-iCAF
ECM-myCAF / TGFβ-myCAF
PODN
MMP2
LRRC15
ACTA2
SDC1
ANTXR1
CASC9
FLRT3
PLXNA2
ADRA2C
HSD17B1
HOXC10
IL32
RBM46
LY75
ADGRG1
KCNQ3
GABRE

Supplementary Figure 1: FAP+ CAF plasticity origin and characterization of primary clusters by flow cytometry

(**A**) UMAP of scRNA-seq dataset from 18 297 FAP+ CAF across 7 BC patients. Colors show the different FAP+ CAF clusters defined by graph-based clustering method, as defined in [1]. (**B**) UMAP showing expression of *PI16* gene. (**C**) *FAP* expression in Detox-iCAF and in normal fibroblasts after pseudo-bulk reconstruction. P-value from Wald test. (**D**) UMAP showing pseudotime computed by Monocle 3 with the Detox-iCAF cluster as the root of the trajectory. **(E, F)** Gating strategy used to characterize primary FAP+ CAF clusters isolated from BC patient samples and cultivated on collagen-coated (**E**) or plastic (**F**) dishes. Representative data of 3 independent experiments. (**G**) Gating strategy used to characterize sorted primary FAP+ CAF from plastic dishes for ECM-myCAF and TGFβ-myCAF and from collagen-coated dishes for Detox-iCAF, IL-iCAF and IFNγ-iCAF. This analysis was performed 1 week after cell sorting to allow cell adaptation and proliferation in the aforementioned conditions. All experiments were performed until passage 6 after sorting to avoid cell senescence. FAP+ CAF cells are gated on FAP+ CD29+ fibroblasts. Among FAP+ CAF cells, ANTXR1 staining is used to distinguish inflammatory iCAF (ANTXR1-) from myofibroblastic myCAF (ANTXR1+). Among ANTXR1+ myCAF, SDC1+ LAMP5- were identified as ECM-myCAF, SDC1+LAMP5+ as TGFβ-myCAF and SDC1- LAMP5- CD9+ as Wound-myCAF. Among ANTXR1-, DLK1, GPC3 and CD74 protein levels were used to identify Detox-iCAF (DLK1+ GPC3+), IL-iCAF (DLK1+ GPC3-) and IFNγ-iCAF (DLK1- GPC3- CD74+). Representative data of 16 independent experiment using 16 primary FAP+ CAF. **(H)** FAP+ CAF cluster characterization from fresh BC patient samples. Cells are gated on Live/Dead-, EPCAM-, CD45-, CD31- and CD235a- to exclude dead, epithelial, hematopoietic, endothelial cells and erythrocytes, respectively. FAP+ CAF clusters were then identified using the same gating strategy as described in (**E, F**). Representative data of 87 patient samples. (**I**) **Left,** Gene Set Enrichment Analysis (GSEA) plots showing the enrichment score (ES) for Detox-iCAF, IL-iCAF and IFNγ-iCAF gene signatures [1] in sorted Detox-iCAF (n = 2), sorted IL-iCAF (n = 2) and sorted IFNγ-iCAF (n = 2) clusters, respectively, compared to sorted ECM-myCAF (n = 3) cluster amplified on plastic dishes. **Right,** Heatmap showing differentially expressed genes between sorted CAF-S1 clusters cultured *in vitro.* In all boxplot the center line, box limits and whiskers indicate the median, upper and lower quartiles and 1.5 × interquartile range.

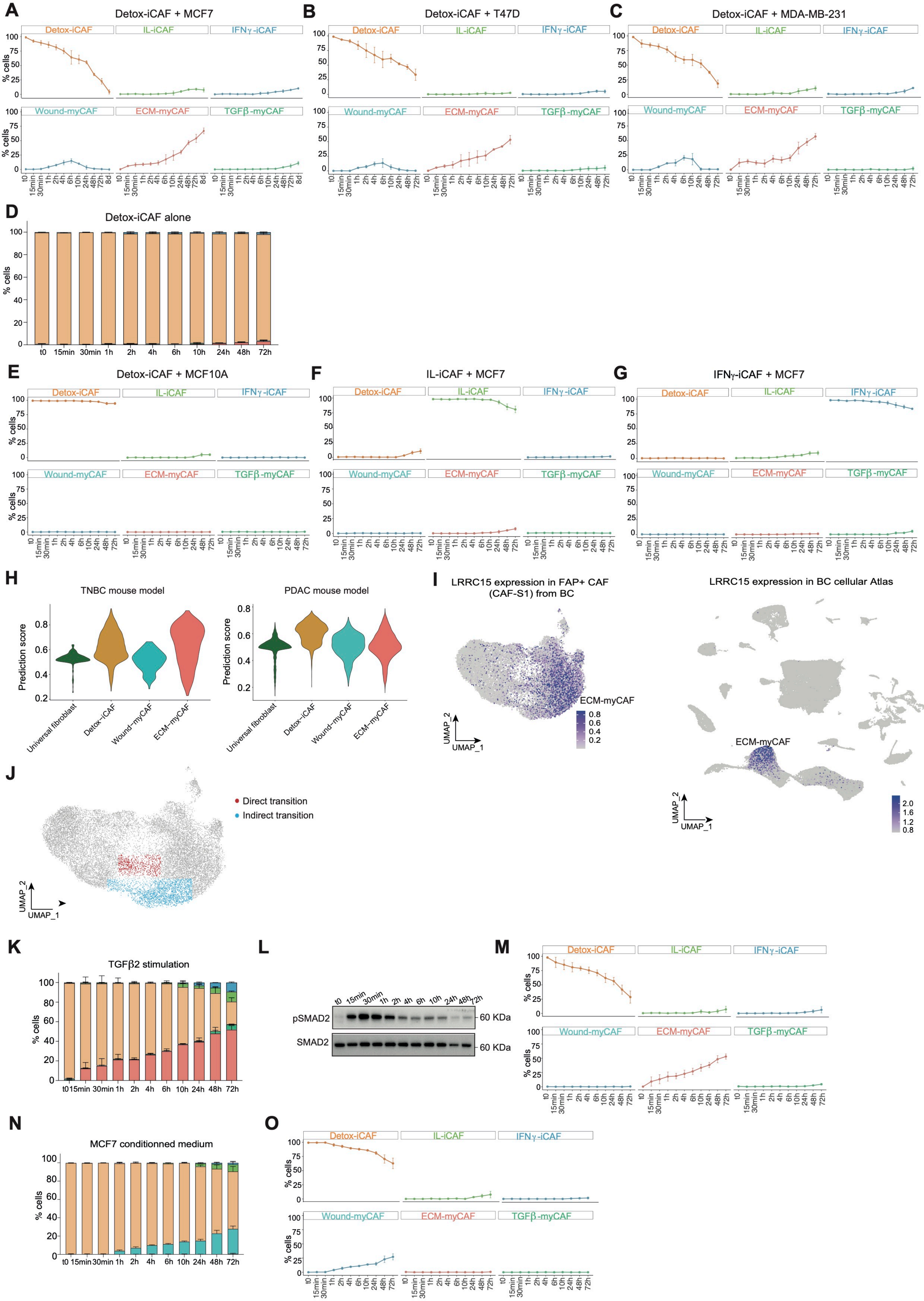

A
Detox-iCAF + MCF7
B
Detox-iCAF + T47D
C
Detox-iCAF + MDA-MB-231
Detox-iCAF
IL-iCAF
IFNγ-iCAF
Wound-myCAF
ECM-myCAF
TGFβ-myCAF
% cells
t0
15min
30min
1h
2h
4h
6h
10h
24h
48h
72h
8d
D
Detox-iCAF alone
E
Detox-iCAF + MCF10A
F
IL-iCAF + MCF7
G
IFNγ-iCAF + MCF7
H
TNBC mouse model
PDAC mouse model
Prediction score
Universal fibroblast
Detox-iCAF
Wound-myCAF
ECM-myCAF
I
LRRC15 expression in FAP+ CAF (CAF-S1) from BC
LRRC15 expression in BC cellular Atlas
UMAP_2
UMAP_1
J
Direct transition
Indirect transition
K
TGFβ2 stimulation
L
pSMAD2
SMAD2
60 KDa
M
N
MCF7 conditionned medium
O

Supplementary Figure 2: Plasticity of FAP+ CAF clusters upon co-culture with cancer cells and TGFβ stimulation

(**A**) Graphs showing the proportions of the different FAP+ CAF clusters among FAP+ CAF at different timepoints of the co-culture of Detox-iCAF with MCF7 cancer cells. Clusters are identified by flow cytometry analyses using FAP+ CAF cluster-specific markers. n = 3 independent experiments. (**B**) Same as in (**A**) for the co-culture of Detox-iCAF with T47D cancer cells. (**C**) Same as in (**A**) for the co-culture of Detox-iCAF with MDA-MB-231 cancer cells. (**D**) Bar blots showing the percentages of the distinct FAP+ CAF clusters after culture of primary Detox-iCAF alone, without cancer cells. Timepoints below each bar plot indicate the duration of the culture. Data are mean ± SEM (n = 3). (**E**) Same as in (**A**) for the co-culture of Detox-iCAF with MCF10A breast epithelial cells. (**F**) Same as in (**A**) for the co-culture of IL-iCAF with MCF7 cancer cells. (**G**) Same as in (**A**) for the co-culture of IFNγ-iCAF with MCF7 cancer cells. (**H**) Violin plots showing prediction scores obtained from label transfer using the BC atlas applied on the BC mouse dataset (**Left**) (n = 3363 cells) and the PDAC mouse dataset (**Right**) (n = 44981 cells). (**I**) UMAP showing *LRRC15* expression in the ECM-myCAF cluster in FAP+ CAF clusters from BC [1] (**Left**) and in the BC scRNAseq cellular atlas built in the current study and shown in **Fig. 3C** (**Right**). (**J**) UMAP of the FAP+ CAF dataset from BC highlighting cells implicated in the direct transition from Detox-iCAF to ECM-myCAF (red) and in the indirect transition from Detox-iCAF to Wound-myCAF (blue). (**K**) Bar plots showing the percentages of each FAP+ CAF cluster after stimulation of the Detox-iCAF cluster (t0) with 10 ng/ml of TGFβ2. Timepoints below each bar plot indicate the duration of the treatment. Data are mean ± SEM (n = 3). (**L**) Representative western blots showing the phosphorylated isoform (P-SMAD2) and the total SMAD2 protein levels after stimulation of the Detox-iCAF cluster (t0) with 10 ng/ml of TGFβ2. Representative data of 3 independent western blots. (**M**) Same as in (**A**) for the stimulation of Detox-iCAF with 10 ng/ml of TGFβ2. (**N**) Same as in (**K**) for incubation of Detox-iCAF with MCF-7-derived conditioned medium. (**O**) Same as in (**A**) for incubation of Detox-iCAF with MCF-7-derived conditioned medium. Source data are provided as a Source Data file.

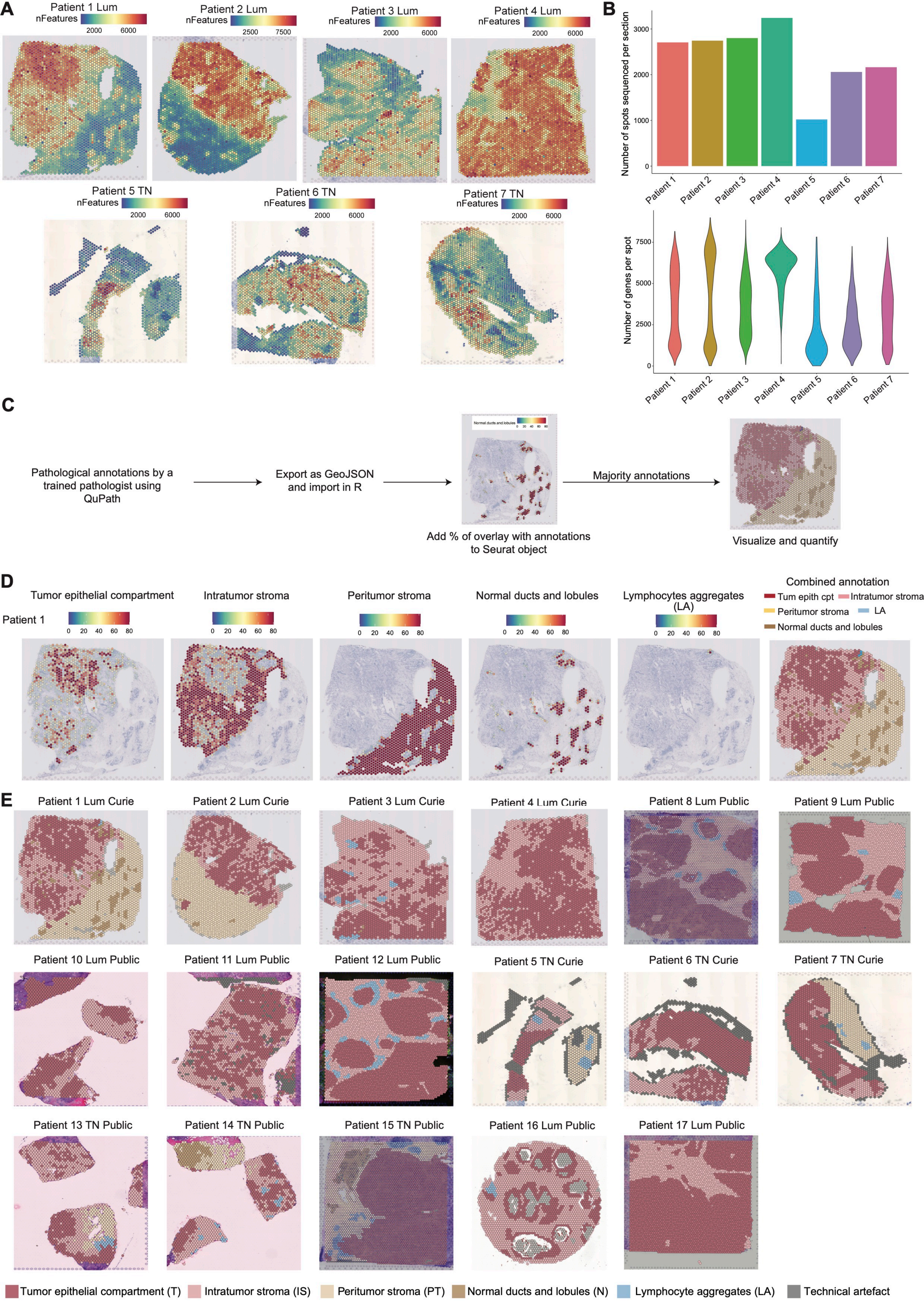
A
Patient 1 Lum
nFeatures
2000 6000
Patient 2 Lum
nFeatures
2500 7500
Patient 3 Lum
nFeatures
2000 6000
Patient 4 Lum
nFeatures
2000 6000
Patient 5 TN
nFeatures
2000 6000
Patient 6 TN
nFeatures
2000 6000
Patient 7 TN
nFeatures
2000 6000
B
Number of spots sequenced per section
0 1000 2000 3000
Patient 1 Patient 2 Patient 3 Patient 4 Patient 5 Patient 6 Patient 7
Number of genes per spot
0 2500 5000 7500
Patient 1 Patient 2 Patient 3 Patient 4 Patient 5 Patient 6 Patient 7
C
Pathological annotations by a trained pathologist using QuPath
Export as GeoJSON and import in R
Normal ducts and lobules
Add % of overlay with annotations to Seurat object
Majority annotations
Visualize and quantify
D
Tumor epithelial compartment
Intratumor stroma
Peritumor stroma
Normal ducts and lobules
Lymphocytes aggregates (LA)
Patient 1
0 20 40 60 80
Combined annotation
Tum epith cpt
Intratumor stroma
Peritumor stroma
LA
Normal ducts and lobules
E
Patient 1 Lum Curie
Patient 2 Lum Curie
Patient 3 Lum Curie
Patient 4 Lum Curie
Patient 8 Lum Public
Patient 9 Lum Public
Patient 10 Lum Public
Patient 11 Lum Public
Patient 12 Lum Public
Patient 5 TN Curie
Patient 6 TN Curie
Patient 7 TN Curie
Patient 13 TN Public
Patient 14 TN Public
Patient 15 TN Public
Patient 16 Lum Public
Patient 17 Lum Public
Tumor epithelial compartment (T)
Intratumor stroma (IS)
Peritumor stroma (PT)
Normal ducts and lobules (N)
Lymphocyte aggregates (LA)
Technical artefact

Supplementary Figure 3: Quality control and pathological annotations of spatial breast cancer sections

(**A**) Number of genes (nFeatures) detected in each spot of the 7 BC sections (4 luminal and 3 triple negative). (**B**) Average number of sequenced spots per section (**Top**) and average number of genes detected (**Bottom**) colored by patient. (**C**) Schematic overview of the workflow allowing the transfer of image-based digital pathologic annotations to the Visium sections for visualization and quantification. Pathological annotation labels were transferred based on a majority area rules, except for tumor epithelial compartment annotation where the label was transferred if more than 30% of the spot covered cancer cells. (**D**) 5 first plots: Visualization of the percentages of overlay between each spot and each pathological annotation in a representative patient (Patient 1). Right plot: Final composite annotations obtained on a majority basis, as detailed in (**C**). (**E**) Visualization of the 5 pathological annotations on the 17 BC Visium sections.

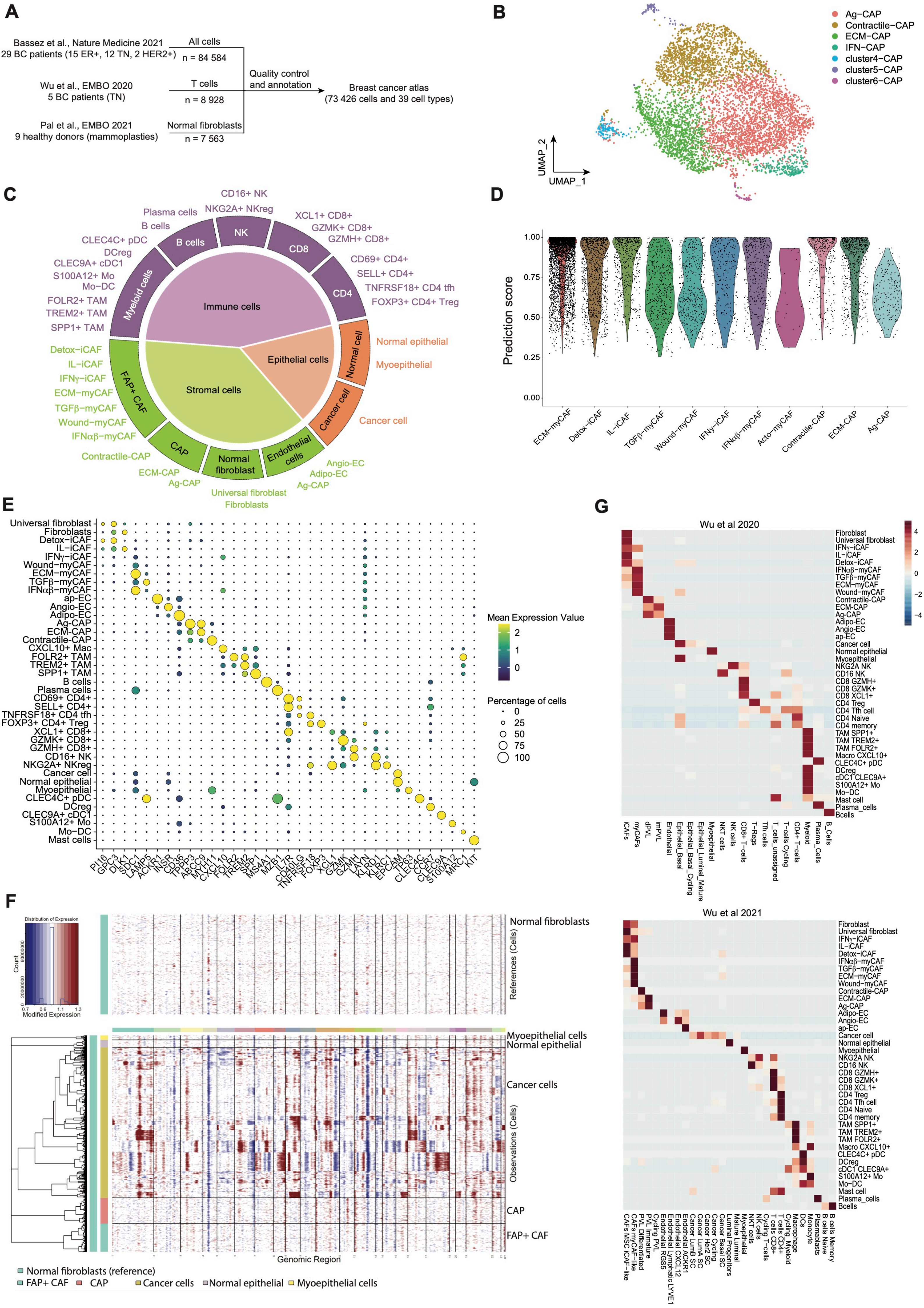
A
Bassez et al., Nature Medicine 2021
29 BC patients (15 ER+, 12 TN, 2 HER2+)
All cells
n = 84 584
Wu et al., EMBO 2020
5 BC patients (TN)
T cells
n = 8 928
Pal et al., EMBO 2021
9 healthy donors (mammoplasties)
Normal fibroblasts
n = 7 563
Quality control
and annotation
Breast cancer atlas
(73 426 cells and 39 cell types)
B
Ag−CAP
Contractile−CAP
ECM−CAP
IFN−CAP
cluster4−CAP
cluster5−CAP
cluster6−CAP
UMAP_2
UMAP_1
C
Immune cells
Epithelial cells
Stromal cells
Myeloid cells
B cells
NK
CD8
CD4
Normal cell
Cancer cell
FAP+ CAF
CAP
Normal fibroblast
Endothelial cells
CD16+ NK
NKG2A+ NKreg
Plasma cells
B cells
XCL1+ CD8+
GZMK+ CD8+
GZMH+ CD8+
CLEC4C+ pDC
DCreg
CLEC9A+ cDC1
S100A12+ Mo
Mo−DC
FOLR2+ TAM
TREM2+ TAM
SPP1+ TAM
CD69+ CD4+
SELL+ CD4+
TNFRSF18+ CD4 tfh
FOXP3+ CD4+ Treg
Normal epithelial
Myoepithelial
Cancer cell
Detox−iCAF
IL−iCAF
IFNγ−iCAF
ECM−myCAF
TGFβ−myCAF
Wound−myCAF
IFNαβ−myCAF
Contractile−CAP
ECM−CAP
Ag−CAP
Universal fibroblast
Fibroblasts
Angio−EC
Adipo−EC
Ag−CAP
D
Prediction score
1.00
0.75
0.50
0.25
0.00
ECM−myCAF
Detox−iCAF
IL−iCAF
TGFβ−myCAF
Wound−myCAF
IFNγ−iCAF
IFNαβ−myCAF
Acto−myCAF
Contractile−CAP
ECM−CAP
Ag−CAP
E
Universal fibroblast
Fibroblasts
Detox−iCAF
IL−iCAF
IFNγ−iCAF
Wound−myCAF
ECM−myCAF
TGFβ−myCAF
IFNαβ−myCAF
ap−EC
Angio−EC
Adipo−EC
Ag−CAP
ECM−CAP
Contractile−CAP
CXCL10+ Mac
FOLR2+ TAM
TREM2+ TAM
SPP1+ TAM
B cells
Plasma cells
CD69+ CD4+
SELL+ CD4+
TNFRSF18+ CD4 tfh
FOXP3+ CD4+ Treg
XCL1+ CD8+
GZMK+ CD8+
GZMH+ CD8+
CD16+ NK
NKG2A+ NKreg
Cancer cell
Normal epithelial
Myoepithelial
CLEC4C+ pDC
DCreg
CLEC9A+ cDC1
S100A12+ Mo
Mo−DC
Mast cells
PI16
GPC3
DLK1
SDC1
LAMP5
ACKR1
INSR
CD36
TPPP3
ABCC9
MYH11
CXCL10
FOLR2
TREM2
SPP1
MS4A1
MZB1
IL7R
CD40LG
TNFRSF18
FOXP3
XCL1
GZMK
GZMH
LAYN
KLRD1
KLRC1
EPCAM
TP63
CLEC4C
CCR7
CLEC9A
S100A12
MRC1
KIT
Mean Expression Value
2
1
0
Percentage of cells
0
25
50
75
100
F
Distribution of Expression
Count
Modified Expression
Normal fibroblasts
References (Cells)
Myoepithelial cells
Normal epithelial
Cancer cells
Observations (Cells)
CAP
FAP+ CAF
Genomic Region
Normal fibroblasts (reference)
FAP+ CAF
CAP
Cancer cells
Normal epithelial
Myoepithelial cells
G
Wu et al 2020
Fibroblast
Universal fibroblast
IFNγ−iCAF
IL−iCAF
Detox−iCAF
IFNαβ−myCAF
TGFβ−myCAF
ECM−myCAF
Wound−myCAF
Contractile−CAP
ECM−CAP
Ag−CAP
Adipo−EC
Angio−EC
ap−EC
Cancer cell
Normal epithelial
Myoepithelial
NKG2A NK
CD16 NK
CD8 GZMH+
CD8 GZMK+
CD8 XCL1+
CD4 Treg
CD4 Tfh cell
CD4 Naive
CD4 memory
TAM SPP1+
TAM TREM2+
TAM FOLR2+
Macro CXCL10+
CLEC4C+ pDC
DCreg
cDC1 CLEC9A+
S100A12+ Mo
Mo−DC
Mast cell
Plasma_cells
Bcells
iCAFs
myCAFs
dPVL
imPVL
Endothelial
Epithelial_Basal
Epithelial_Basal_Cycling
Epithelial_Luminal_Mature
Myoepithelial
NKT cells
NK cells
CD8+ T−cells
T−Regs
Tfh cells
T_cells_unassigned
T−cells Cycling
CD4+ T−cells
Myeloid
Plasma_Cells
B_Cells
4
2
0
−2
−4
Wu et al 2021
Fibroblast
Universal fibroblast
IFNγ−iCAF
IL−iCAF
Detox−iCAF
IFNαβ−myCAF
TGFβ−myCAF
ECM−myCAF
Wound−myCAF
Contractile−CAP
ECM−CAP
Ag−CAP
Adipo−EC
Angio−EC
ap−EC
Cancer cell
Normal epithelial
Myoepithelial
NKG2A NK
CD16 NK
CD8 GZMH+
CD8 GZMK+
CD8 XCL1+
CD4 Treg
CD4 Tfh cell
CD4 Naive
CD4 memory
TAM SPP1+
TAM TREM2+
TAM FOLR2+
Macro CXCL10+
CLEC4C+ pDC
DCreg
cDC1 CLEC9A+
S100A12+ Mo
Mo−DC
Mast cell
Plasma_cells
Bcells
CAFs MSC iCAF−like
CAFs myCAF−like
PVL Differentiated
PVL Immature
Cycling PVL
Endothelial RGS5
Endothelial Lymphatic LYVE1
Endothelial CXCL12
Endothelial ACKR1
Cancer LumB SC
Cancer LumA SC
Cancer Her2 SC
Cancer Cycling
Cancer Basal SC
Luminal Progenitors
Mature Luminal
Myoepithelial
NKT cells
NK cells
Cycling T−cells
T cells CD8+
T cells CD4+
Cycling_Myeloid
Macrophage
DCs
Monocyte
Plasmablasts
B cells Naive
B cells Memory

Supplementary Figure 4: Building of a high-resolution breast cancer cellular atlas

(**A**) Publicly available scRNA-seq datasets [2, 3, 4] used for the construction of a human BC cellular atlas that include 73 426 cells from 34 BC patients and 9 healthy donors. (**B**) UMAP of 5716 CAP across 3 BC patients allowing the visualization of 7 clusters. Colors show the different CAP clusters defined by graph-based clustering. (**C**) Schematic representation of the 39 different cell types and states defined in the BC cellular atlas. (**D**) Violin plots showing the prediction score obtained after Label transfer for the annotation of FAP+ CAF and CAP cells from the BC atlas using previously published FAP+ CAF cells [1] and newly generated CAP from (**B**). (**E**) Expression values of specific genes of the 39 cell types and states from the BC atlas. Colors show mean expression level of each gene in each cell type or state. Sizes of circles represent the percentage of cells expressing one given gene. (**F**) Heatmap showing inferred copy number variation (CNV) for epithelial cells and CAF. scRNA-seq data from normal fibroblasts were used as reference for normalization and are shown in the upper part. Each row corresponds to one cell clustered by cell type, and each column to genes ordered by chromosome position. Amplifications (red) and deletions (blue) are inferred from the gene expression. (**G**) Heatmap showing the correspondences between original cell type annotations from 2 published BC scRNA-seq datasets [3] (**Top**) and [5] (**Bottom**) in columns and predicted annotations from Label transfer using BC atlas as reference in rows. Values are centered and scaled in row.

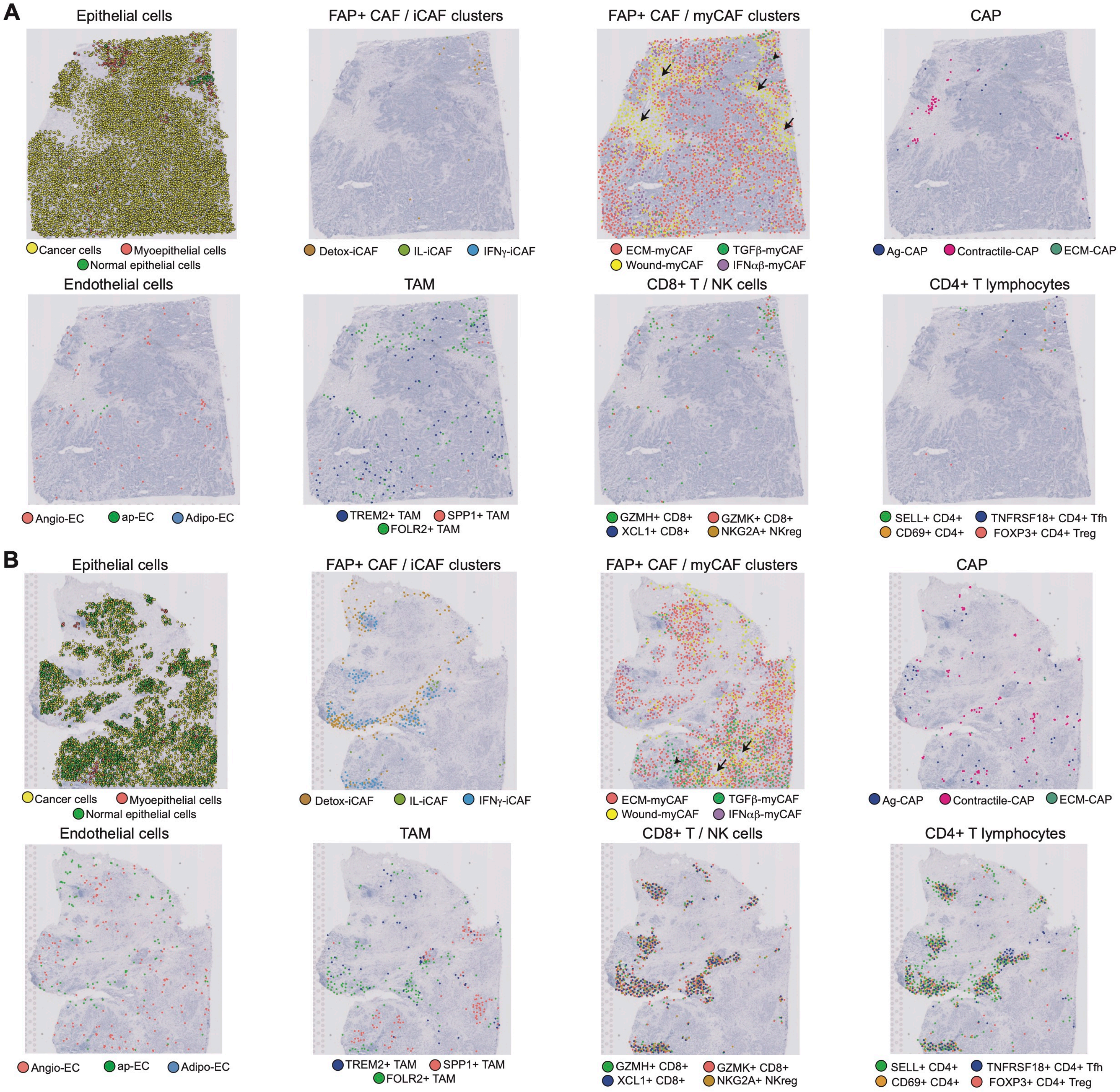

A
Epithelial cells
Cancer cells
Myoepithelial cells
Normal epithelial cells
FAP+ CAF / iCAF clusters
Detox-iCAF
IL-iCAF
IFNγ-iCAF
FAP+ CAF / myCAF clusters
ECM-myCAF
TGFβ-myCAF
Wound-myCAF
IFNαβ-myCAF
CAP
Ag-CAP
Contractile-CAP
ECM-CAP
Endothelial cells
Angio-EC
ap-EC
Adipo-EC
TAM
TREM2+ TAM
SPP1+ TAM
FOLR2+ TAM
CD8+ T / NK cells
GZMH+ CD8+
GZMK+ CD8+
XCL1+ CD8+
NKG2A+ NKreg
CD4+ T lymphocytes
SELL+ CD4+
TNFRSF18+ CD4+ Tfh
CD69+ CD4+
FOXP3+ CD4+ Treg
B
Epithelial cells
Cancer cells
Myoepithelial cells
Normal epithelial cells
FAP+ CAF / iCAF clusters
Detox-iCAF
IL-iCAF
IFNγ-iCAF
FAP+ CAF / myCAF clusters
ECM-myCAF
TGFβ-myCAF
Wound-myCAF
IFNαβ-myCAF
CAP
Ag-CAP
Contractile-CAP
ECM-CAP
Endothelial cells
Angio-EC
ap-EC
Adipo-EC
TAM
TREM2+ TAM
SPP1+ TAM
FOLR2+ TAM
CD8+ T / NK cells
GZMH+ CD8+
GZMK+ CD8+
XCL1+ CD8+
NKG2A+ NKreg
CD4+ T lymphocytes
SELL+ CD4+
TNFRSF18+ CD4+ Tfh
CD69+ CD4+
FOXP3+ CD4+ Treg

Supplementary Figure 5: Spatial organization of breast cancer micro-environment

(**A, B**) Deconvolution results based on the scRNA-seq cell atlas showed in **Fig. 3B** displayed on two representative BC sections. Each colored dot corresponds to a single cell and the different colors show distinct cell types and states.

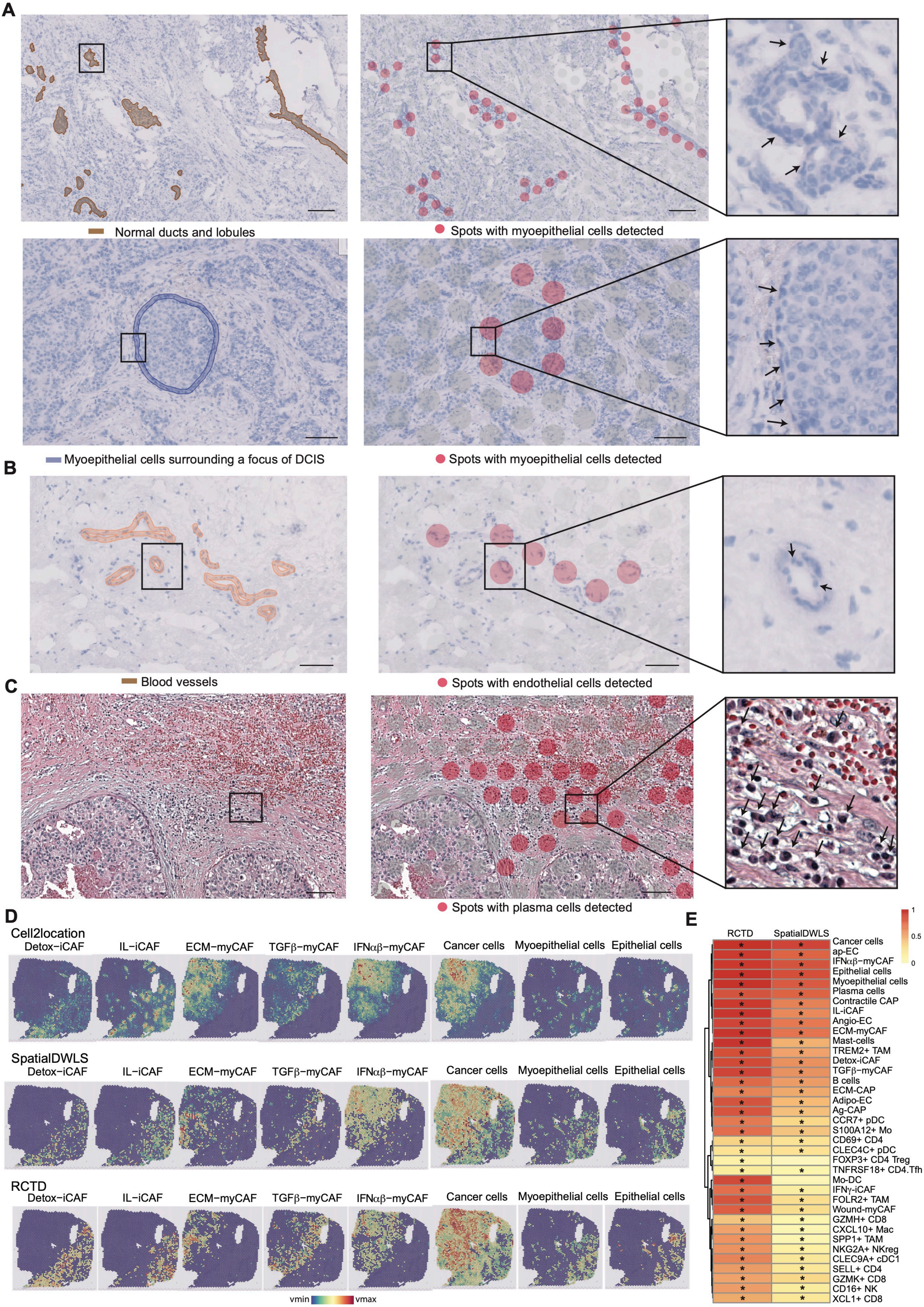

A
Normal ducts and lobules
Spots with myoepithelial cells detected
Myoepithelial cells surrounding a focus of DCIS
Spots with myoepithelial cells detected
B
Blood vessels
Spots with endothelial cells detected
C
Spots with plasma cells detected
D
Cell2location
Detox−iCAF
IL−iCAF
ECM−myCAF
TGFβ−myCAF
IFNαβ−myCAF
Cancer cells
Myoepithelial cells
Epithelial cells
SpatialDWLS
RCTD
vmin
vmax
E
RCTD
SpatialDWLS
Cancer cells
ap-EC
IFNαβ−myCAF
Epithelial cells
Myoepithelial cells
Plasma cells
Contractile CAP
IL-iCAF
Angio-EC
ECM-myCAF
Mast-cells
TREM2+ TAM
Detox-iCAF
TGFβ-myCAF
B cells
ECM-CAP
Adipo-EC
Ag-CAP
CCR7+ pDC
S100A12+ Mo
CD69+ CD4
CLEC4C+ pDC
FOXP3+ CD4 Treg
TNFRSF18+ CD4.Tfh
Mo-DC
IFNγ-iCAF
FOLR2+ TAM
Wound-myCAF
GZMH+ CD8
CXCL10+ Mac
SPP1+ TAM
NKG2A+ NKreg
CLEC9A+ cDC1
SELL+ CD4
GZMK+ CD8
CD16+ NK
XCL1+ CD8
1
0.5
0

Supplementary Figure 6: Accuracy of cell2location deconvolution compared to pathology morphological annotations

(**A**) **Left**, Representative H&E images annotated by pathologists based on morphological features showing normal ducts and lobules (**Top**), and myoepithelial cells surrounding a DCIS focus (**Bottom**). **Middle**, Same section analyzed by spatial transcriptomics identifying myoepithelial cells after deconvolution by cell2location. Scale bars: 200µm (**Top**) and 100µm (**Bottom**). **Right**, Higher magnification of the same area shown in the Middle. Representative image of 17 sections. (**B**) **Left**, Representative image showing presence of blood vessels identified by pathologists. **Middle** and Right, Same section analyzed by spatial transcriptomics identifying endothelial cells after deconvolution. Scale bar = 100µm. Representative image of 17 sections. (**C**) Same as (**B**) for plasma cells. Scale bar = 100µm. Arrows in the magnification panels underline myoepithelial, endothelial and plasma cells under spots where the cell types were detected. Representative image of 17 sections. (**D**) Comparison of deconvolution results of the section displayed in **Fig. 3C** (representative of the main pathological compartments) for the different cell types and states by using two other deconvolution methods (RCTD and SpatialDWLS). Cell2location values represent the number of deconvoluted cells per spot. RCTD and SpatialDWLS results are expressed as cell type proportions per spot. (**E**) Data show correlations between Cell2location deconvolution and RCTD or SpatialDWLS methods. The symbol * indicates significant ($P < 0.05$) correlations from Pearson correlation test. Colors show correlation coefficients.

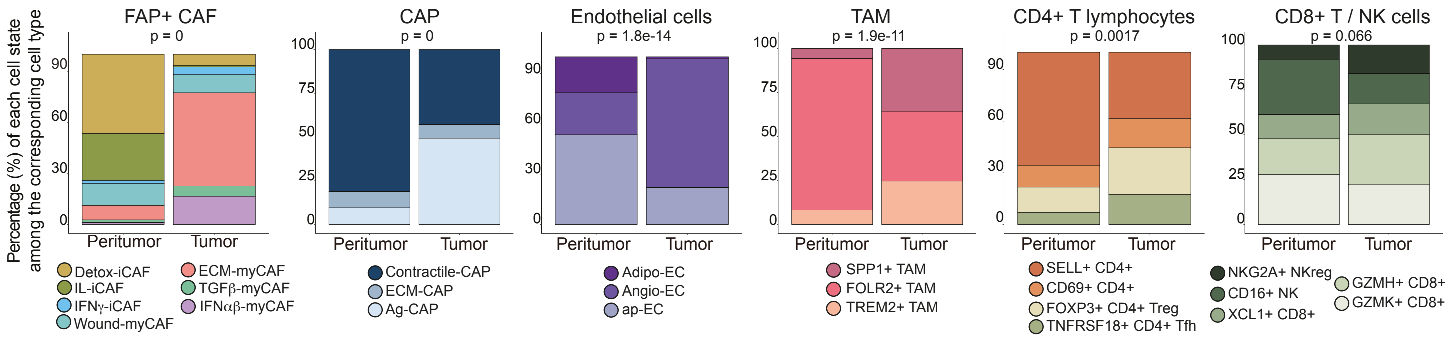

Supplementary Figure 7: Proportion of each cell state between peritumor and tumor compartments

Mean proportion of each cell state between the peritumor and the tumor bed (Tumor epithelial compartment and intratumor stroma) among total FAP+ CAF, CAP, endothelial cells, TAM, CD4+ T lymphocytes and CD8+ T cells and NK cells (N = 17 BC sections for Tumor and N = 7 for Peritumor). P-values from Fisher's exact test.

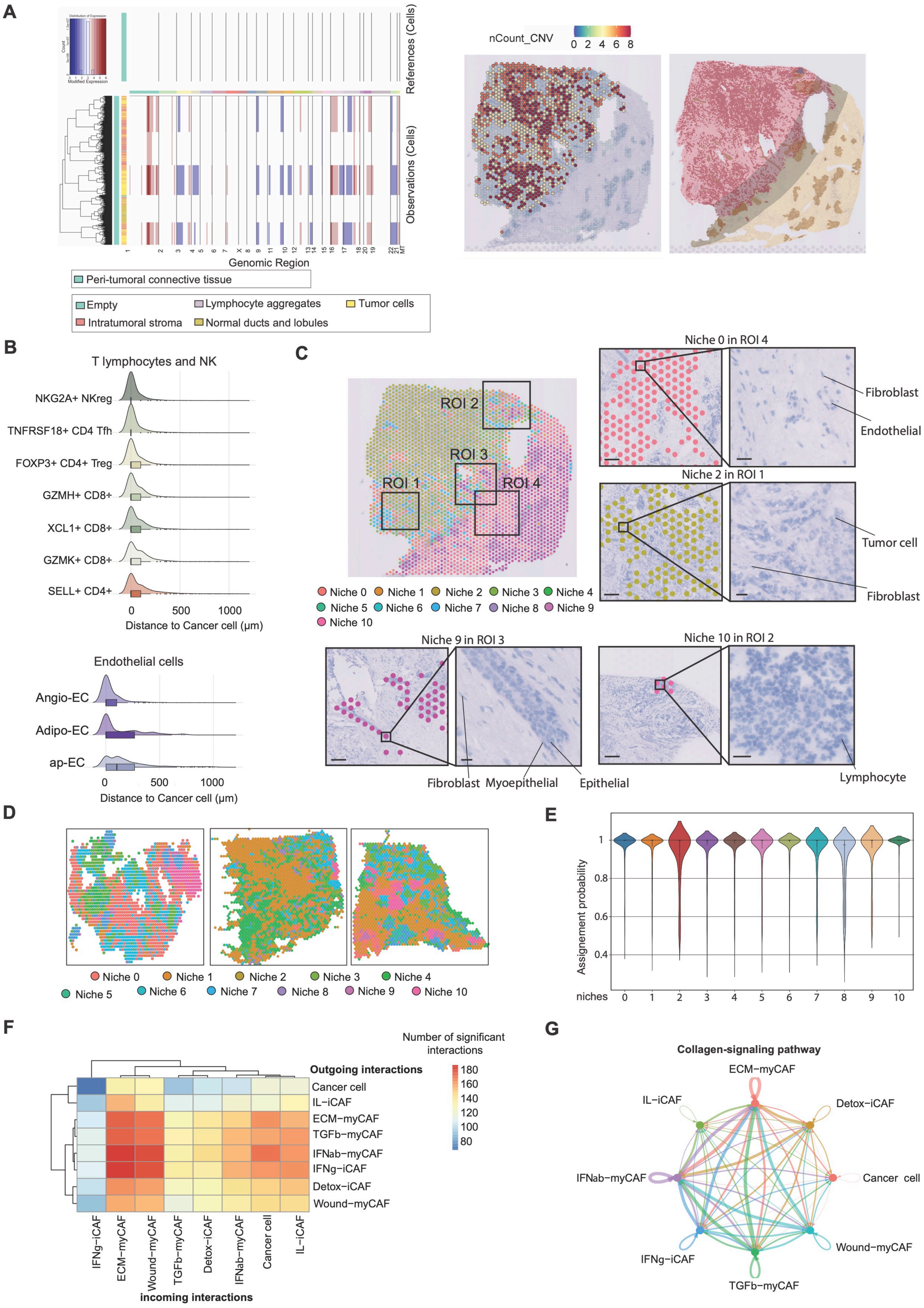
A
References (Cells)
Observations (Cells)
Genomic Region
Peri-tumoral connective tissue
Empty
Lymphocyte aggregates
Tumor cells
Intratumoral stroma
Normal ducts and lobules
nCount_CNV
B
T lymphocytes and NK
NKG2A+ NKreg
TNFRSF18+ CD4 Tfh
FOXP3+ CD4+ Treg
GZMH+ CD8+
XCL1+ CD8+
GZMK+ CD8+
SELL+ CD4+
Distance to Cancer cell (μm)
Endothelial cells
Angio-EC
Adipo-EC
ap-EC
Distance to Cancer cell (μm)
C
ROI 1
ROI 2
ROI 3
ROI 4
Niche 0 in ROI 4
Fibroblast
Endothelial
Niche 2 in ROI 1
Tumor cell
Fibroblast
Niche 9 in ROI 3
Niche 10 in ROI 2
Fibroblast
Myoepithelial
Epithelial
Lymphocyte
D
E
Assignement probability
niches
F
Number of significant interactions
Outgoing interactions
Cancer cell
IL−iCAF
ECM−myCAF
TGFb−myCAF
IFNab−myCAF
IFNg−iCAF
Detox−iCAF
Wound−myCAF
incoming interactions
G
Collagen-signaling pathway
ECM−myCAF
IL−iCAF
Detox−iCAF
IFNab−myCAF
Cancer cell
IFNg−iCAF
Wound−myCAF
TGFb−myCAF

Supplementary Figure 8: Cell-cell distances and communications in breast cancer micro-environment

(A) Left, scRNA-seq derived CNV analysis for each spatial transcriptomic barcoded spot from patient 1, which contained a mixture of stroma and cancer cells. Spot annotated as peritumoral connective tissue were used as reference (red, gain; blue, loss). **Right**, number of CNV detected per spot on the section and matching pathological annotation on the same section. (**B**) Distribution of T lymphocytes, NK and endothelial cells (EC) populations according to the distance to cancer cells (in µm). Distances are computed between the closest cancer cell obtained by deconvolution and T lymphocytes, NK and EC**.** Cell types are ranked based on their median distance to cancer cells. (**C**) Section displayed in **Fig.3C**. Colors indicate the niches. Black boxes correspond to representative areas (Regions of Interest, ROI) for 4 selected niches shown at 2 different magnifications on the Bottom and on the Right. For each ROI: **Left**: Spots indicating the niche; Scale bar = 150 µm.: **Right**: Corresponding histological section of the niche; Scale bar = 25 µm. (**D**) Predicted BC cellular niches on an external cohort of BC sections [6] analyzed by spatial transcriptomics. (**E**) Assignment probability of the niches for the spots from the external cohort. (**F**) Number of significant interactions between FAP+ CAF clusters and cancer cells inferred by CellChat. The direction of interactions is represented from the cell type indicated in rows (outgoing interactions) to the cell type indicated in columns (incoming interactions). (**G**) Chord diagram showing inferred Collagen-signaling pathway between FAP+ CAF clusters and cancer cells. Edges width represents the communication probability computed by CellChat, colored by cell type. In all boxplot the center line, box limits and whiskers indicate the median, upper and lower quartiles and 1.5 × interquartile range.

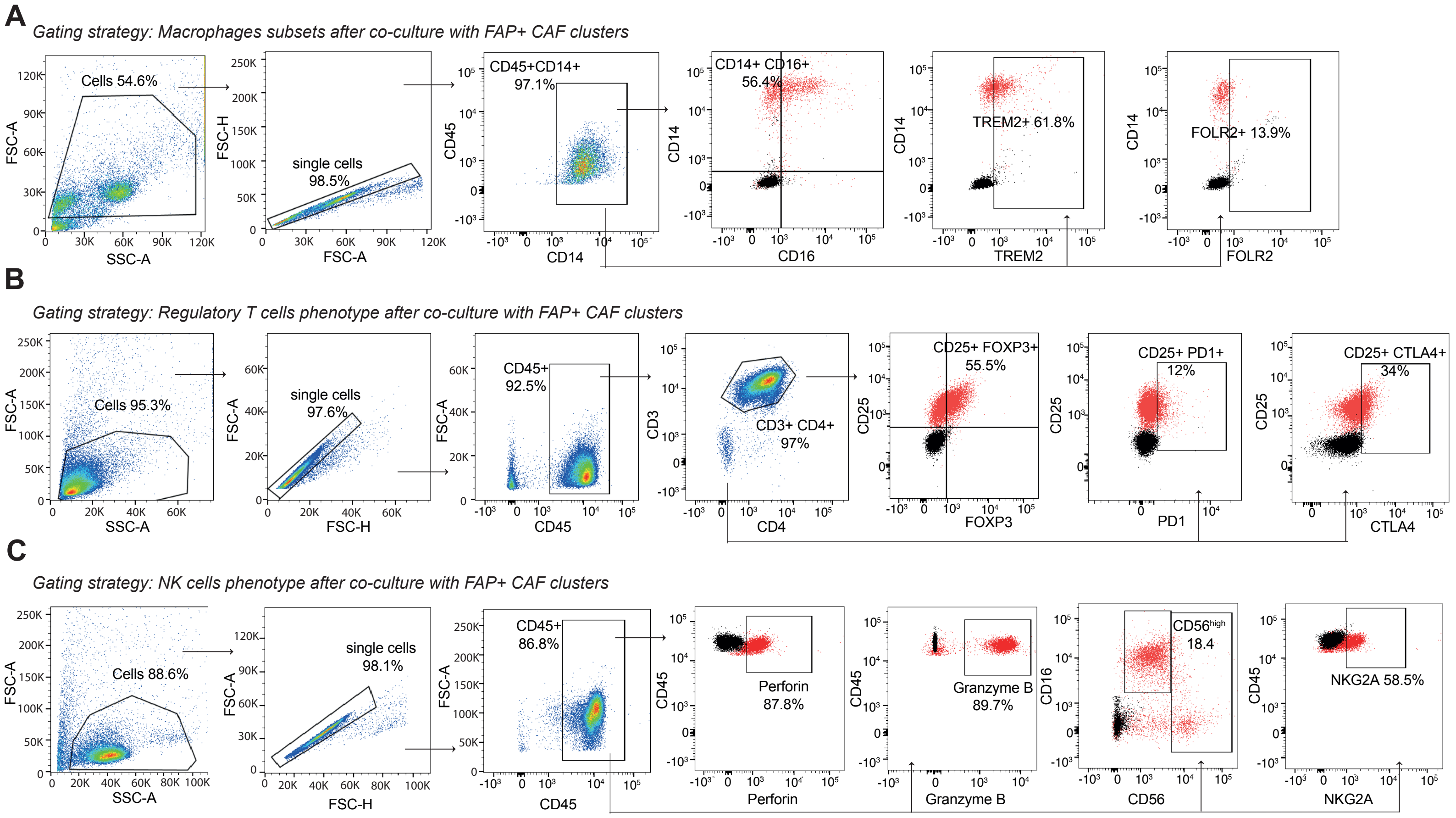

A
Gating strategy: Macrophages subsets after co-culture with FAP+ CAF clusters
Cells 54.6%
FSC-A
SSC-A
FSC-H
single cells
98.5%
FSC-A
CD45+CD14+
97.1%
CD45
CD14
CD14+ CD16+
56.4%
CD14
CD16
TREM2+ 61.8%
CD14
TREM2
FOLR2+ 13.9%
CD14
FOLR2
B
Gating strategy: Regulatory T cells phenotype after co-culture with FAP+ CAF clusters
Cells 95.3%
FSC-A
SSC-A
single cells
97.6%
FSC-A
FSC-H
CD45+
92.5%
FSC-A
CD45
CD3
CD3+ CD4+
97%
CD4
CD25+ FOXP3+
55.5%
CD25
FOXP3
CD25+ PD1+
12%
CD25
PD1
CD25+ CTLA4+
34%
CD25
CTLA4
C
Gating strategy: NK cells phenotype after co-culture with FAP+ CAF clusters
Cells 88.6%
FSC-A
SSC-A
single cells
98.1%
FSC-A
FSC-H
CD45+
86.8%
FSC-A
CD45
Perforin
87.8%
CD45
Perforin
Granzyme B
89.7%
CD45
Granzyme B
CD56high
18.4
CD16
CD56
NKG2A 58.5%
CD45
NKG2A

Supplementary Figure 9: Gating strategies used to characterize isolated immune cells after co-culture with FAP+ CAF clusters *in vitro*

(**A**) Gating Strategy used to characterize TAM subsets after 24h of co-culture with FAP+ CAF clusters. Cells were first gated based on their size (FSC-A) and granularity (SSC-A). Myeloid cells were identified as CD45+ CD14+ and their phenotype was assessed by flow cytometry for CD16, TREM2 and FOLR2 protein levels. Representative data of 9 independent experiments. (**B**) Gating strategy used to characterize regulatory T cells among total CD4+ CD3+ T lymphocytes after 16h of co- culture with FAP+ CAF clusters. Cells were first gated based on their size (FSC-A) and granularity (SSC-A). Among CD45+ CD3+ CD4+ cells, regulatory T lymphocytes were identified based on CD25 and FOXP3 protein levels. The percentage of CTLA4 as well as PD1 were then evaluated. Representative data of 8 independent experiments. (**C**) Gating strategy used to characterize NK cells after 24h of co-culture with FAP+ CAF clusters. Cells were first gated based on their size (FSC-A) and granularity (SSC-A). Among CD45+ hematopoietic cells, the percentage of NKG2A as well as granzyme B and perforin levels were evaluated. NK cells were also separated in two subsets according to CD56 and CD16 protein levels and defined as cytotoxic NK ($CD16^{high}$ $CD56^{Med}$) and noncytotoxic NK ($CD16^{-}$ $CD56^{high}$). Representative data of 7 independent experiments.

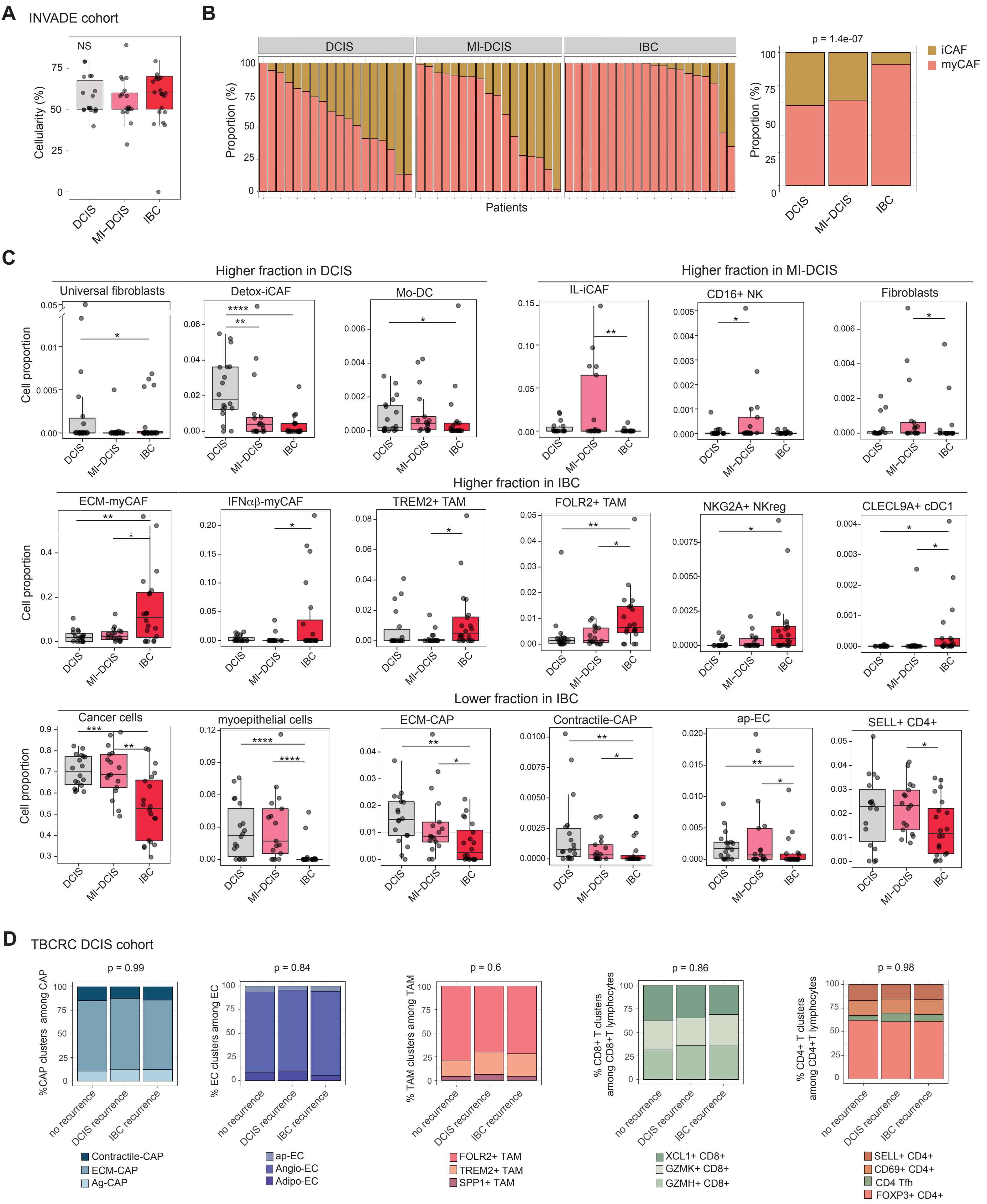

A
INVADE cohort
NS
Cellularity (%)
DCIS
MI-DCIS
IBC
B
DCIS
MI-DCIS
IBC
Proportion (%)
Patients
p = 1.4e-07
iCAF
myCAF
C
Higher fraction in DCIS
Universal fibroblasts
Detox-iCAF
Mo-DC
Higher fraction in MI-DCIS
IL-iCAF
CD16+ NK
Fibroblasts
Cell proportion
Higher fraction in IBC
ECM-myCAF
IFNαβ-myCAF
TREM2+ TAM
FOLR2+ TAM
NKG2A+ NKreg
CLECL9A+ cDC1
Lower fraction in IBC
Cancer cells
myoepithelial cells
ECM-CAP
Contractile-CAP
ap-EC
SELL+ CD4+
D
TBCRC DCIS cohort
p = 0.99
%CAP clusters among CAP
p = 0.84
% EC clusters among EC
p = 0.6
% TAM clusters among TAM
p = 0.86
% CD8+ T clusters among CD8+T lymphocytes
p = 0.98
% CD4+ T clusters among CD4+T lymphocytes
no recurrence
DCIS recurrence
IBC recurrence
Contractile-CAP
ECM-CAP
Ag-CAP
ap-EC
Angio-EC
Adipo-EC
FOLR2+ TAM
TREM2+ TAM
SPP1+ TAM
XCL1+ CD8+
GZMK+ CD8+
GZMH+ CD8+
SELL+ CD4+
CD69+ CD4+
CD4 Tfh
FOXP3+ CD4+

Supplementary Figure 10: Deconvolution of bulk RNA-seq from retrospective BC cohorts

(**A-C**) Data from the Invade BC patient cohort (N = 55). (**A**) Boxplot showing percentage of tumor cellularity across DCIS (N = 18), MI-DCIS (N = 17) and IBC (N = 20) samples. P-value from Welch's t-test. (**B**) Bar plots representing the relative composition in iCAF and myCAF cells among total FAP+ CAF cells per patient (**Left**) or according to BC invasive status (DCIS, MI-DCIS and IBC) (**Right**). P-value from Fisher's exact test. (**C**) Boxplots of the relative cell type composition across DCIS (N = 18), MI-DCIS (N = 17) and IBC (N = 20) samples. Cell types with significant differences between BC invasive status are represented. P-values from Mann-Whitney test. Symbols for significance: *, p <0.05, **, p <0.01; ***, p <0.001; ****, p <0.0001. (**D**) Data from the TBCRC cohort of DCIS patients (N = 216 patients, N = 66 with DCIS recurrence, N = 55 with IBC recurrence and N = 95 without recurrence). Bar plots showing the relative proportions of clusters among CAP, endothelial, myeloid and lymphoid cells according to recurrence. P-values from Fisher's exact test. In all boxplot the center line, box limits and whiskers indicate the median, upper and lower quartiles and 1.5 × interquartile range.

Supplementary Table 1

		Prospective Cohort 1 (FACS analysis)	Prospective Cohort 2 (primary cell lines)
**Number of patients**		84	16
**Age at diagnosis**			
	**< 50 years**	28 (33.3%)	8 (50%)
	**> or = 50 years**	56 (66.7%)	8 (50%)
**Multifocality**			
	**no**	51 (60.7%)	7 (43.7%)
	**yes**	31 (37%)	9 (56.3%)
	**NA**	2 (2.3%)	0
**Histological type**			
	**ductal**	54 (64.3%)	12 (75%)
	**lobular**	16 (19%)	2 (12.5%)
	**other**	14 (16.7%)	2 (12.5%)
**Grade**			
	**G1**	12 (14.3%)	2 (12.5%)
	**G2**	45 (53.6%)	10 (62.5%)
	**G3**	27 (32.1%)	4 (25%)
	**NA**	0	0
**Tumor size**			
	**pT1**	35 (41.7%)	5 (31.1%)
	**pT2**	41 (48.8%)	11 (68.9%)
	**pT3**	8 (9.5%)	0
	**NA**	0	0
**Lymph node metastases**			
	**pN0**	56 (66.7%)	7 (43.7%)
	**pN1**	20 (23.8%)	6 (37.5%)
	**pN2**	4 (4.7%)	2 (12.5%)
	**pN3**	2 (2.4%)	1 (6.3%)
	**NA**	2 (2.4%)	0
**ER status**			
	**ER-**	5 (6%)	0
	**ER+**	79 (94%)	16 (100%)
**PR status**			
	**PR-**	15 (17.8%)	3 (18.75%)
	**PR+**	69 (82.2%)	13 (81.25%)
**HER2 status**			
	**Negative**	81 (96.4%)	15 (93.75%)
	**Positive**	3 (3.6%)	1 (6.25%)
**HistoMolecular class**			
	**LumA**	45 (53.6%)	9 (56.25%)
	**LumB**	33 (39.4%)	6 (37.5%)
	**LumB /HER2**	3 (3.5%)	1 (6.25)
	**TN**	3 (3.5%)	0
**Surgery**			
	**Mastectomy**	46 (54.7%)	6 (37.5%)
	**Lumpectomy**	38 (45.3%)	10 (62.5%)
**Radiotherapy**			
	**Yes**	60 (71.5%)	14 (87.5%)
	**No**	16 (19%)	2 (12.5%)
	**NA**	8 (9.5%)	0
**Hormonotherapy**			
	**Yes**	67 (79.8%)	15 (93.75%)
	**No**	10 (12%)	1 (6.25%)
	**NA**	7 (8.2%)	0
**Adjuvant chemotherapy**			
	**Yes**	40 (47.5%)	11 (68.75%)
	**No**	36 (43%)	5 (31.25%)
	**NA**	8 (9.5%)	0

Supplementary Table 1: Description of the prospective cohorts used in this study

Two prospective cohorts have been studied here. Tumor samples from cohort 1 have been used for multicolor flow cytometry and spatial transcriptomics. Tumor samples from cohort 2 have been used for culture of primary FAP+ CAF clusters. Abbreviations: pT: Pathological tumor size; pN: Pathological lymph node status; ER: Estrogen receptor; PR: Progesterone receptor; LumA: Luminal A; LumB: Luminal B; TN: Triple Negative; HER2: Human epidermal growth factor receptor 2; NA: Not available.

Supplementary Table 2

		ALL	DCIS	MI-DCIS	IBC
**Number of patients**		55	18	17	20
**Sex**					
	**Female**	55	18	17	20
**Age at diagnosis**					
	**min-max (median)**	23 - 84 (50)	24 - 56 (44.5)	23 - 70 (56)	33 - 84 (53)
**TILs**					
	**< 10%**	11 (20%)	9 (50%)	2 (11.8%)	
	**10-30%**	18 (32.7%)	7 (38.9%)	11 (64.7%)	
	**> 30%**	6 (10.9%)	2 (11.1%)	4 (23.5%)	
	**NA**	20 (36.4%)			20 (100%)
**Grade (DCIS and MI-DCIS)**					
	**low**	2 (5.7%)	2 (11.1%)		
	**Intermediate**	10 (28.6%)	6 (33.3%)	4 (23.5%)	
	**High**	23 (65.7%)	10 (55.6%)	13 (76.5%)	
**Grade (IBC)**					
	**1**	9 (45%)			9 (45%)
	**2**	3 (15%)			3 (15%)
	**3**	8 (40%)			8 (40%)
**pT (UICC)**					
	**pT1**	9 (16.4%)			9 (45%)
	**pT2**	11 (20%)			11 (55%)
	**pTis**	18 (32.7%)	18 (100%)		
	**pT1Mi**	17 (30.9%)		17 (100%)	
**pN (UICC)**					
	**pN0**	34 (61.8%)	11 (61.1%)	12 (70.6%)	11 (55%)
	**pN1**	9 (16.4%)	1 (5.6%)	1 (5.9%)	7 (35%)
	**pN2**	2 (3.6%)			2 (10%)
	**NA**	10 (18.2%)	6 (33.3%)	4 (23.5%)	
**ER status**					
	**ER-**	18 (32.7%)	4 (22.2%)	9 (52.9%)	5 (25%)
	**ER+**	37 (67.3%)	14 (77.8%)	8 (47.1%)	15 (75%)
**PR status**					
	**PR-**	26 (47.3%)	7 (38.9%)	12 (70.6%)	7 (35%)
	**PR+**	29 (52.7%)	11 (61.1%)	5 (29.4%)	13 (65%)
**HER2 status**					
	**Negative**	41 (74.5%)	16 (88.9%)	9 (52.9%)	16 (80%)
	**Positive**	14 (25.5%)	2 (11.1%)	8 (47.1%)	4 (20%)
**HistoMolecular class**					
	**LumA**	27 (49.1%)	11 (61.1%)	6 (35.3%)	10 (50%)
	**LumB**	9 (16.4%)	3 (16.7%)	2 (11.8%)	4 (20%)
	**HER2**	11 (20%)	1 (5.6%)	7 (41.2%)	3 (15%)
	**LumB /HER2**	3 (5.5%)	1 (5.6%)	1 (5.9%)	1 (5%)
	**TN**	5 (9.1%)	2 (11.1%)	1 (5.9%)	2 (10%)
**Surgery**					
	**Mastectomy**	31 (56.4%)	12 (66.7%)	7 (41.2%)	12 (60%)
	**Lumpectomy**	24 (43.6%)	6 (33.3%)	10 (58.8%)	8 (40%)
**Radiotherapy**					
	**Yes**	33 (60%)	7 (38.9%)	11 (64.7%)	15 (75%)
	**No**	22 (40%)	11 (61.1%)	6 (35.3%)	5 (25%)
**Hormonotherapy**					
	**Yes**	16 (29.1%)	1 (5.6%)	1 (5.9%)	14 (70%)
	**No**	39 (70.9%)	17 (94.4%)	16 (94.1%)	6 (30%)
**Adjuvant chemotherapy**					
	**Yes**	11 (20%)	1 (5.6%)		10 (50%)
	**No**	44 (80%)	17 (94.4%)	17 (100%)	10 (50%)

Supplementary Table 2: Description of the retrospective INVADE cohort

Retrospective BC cohort of 55 patients suffering from pre-invasive Ductal Carcinoma in Situ (DCIS) lesions (N = 18), micro-invasive DCIS (MI-DCIS) corresponding to DCIS lesions with invasive foci not exceeding 1 mm (N = 17) and Invasive Breast Cancer (IBC) (N = 20). Abbreviations: TILs: Tumor infiltrating lymphocytes; pT: Pathological tumor size; pN: Pathological lymph node status; ER: Estrogen receptor; PR: Progesterone receptor; LumA: Luminal A; LumB: Luminal B; TN: Triple Negative; HER2: Human epidermal growth factor receptor 2; NA: Not available.

Supplementary Table 3

Antibodies for CAF-S1 clusters characterization	Reference	Dilution	Clone
Brilliant Violet 605 anti-human CD326 (EpCAM)	BioLegend, #324224	1/50	9C4
PE/Cyanine7 anti-human CD31 antibody	BioLegend, #303118	1/100	WM59
BUV395 Anti-human CD45	BD Biosciences, #563792	1/50	HI30
PerCP/Cyanine5.5 anti-human CD235a	Biolegend, #349110	1/50	HI264
Human Fibroblast Activation Proetin Alpha/FAP Antibody	R&D Systems, #MAB3715	1/100	427819
Alexa Fluor700 anti-human CD29	BioLegend, #303020	1/100	TS2/16
Alexa Fluor 405 TEM8/ANTXR1 antibody	Novus Biologicals #NB100-56585AF405	1/25	200C1339(SB20)
BUV737 anti human CD138 (SDC1)	BD Biosciences #612834	1/25	MI15
Human Glypican 3 Alexa Fluor 594 Antibody	R&D systems #FAB2119T	1/20	307801
Human DLK1 Alex Fluor 488 Antibody	R&D systems #FAB1144G	1/20	211309
BV711 Anti-human CD9	BD Biosciences #743050	1/100	M-L13
BV786 Anti-human CD74	BD Biosciences #743736	1/100	LN2
PE anti-human LAMP5 antbody	Miltenyi Biotec #130-109-156	1/10	REA590
Fluorescent dye Zenon APC Mouse IgG1 labeling kit	Thermo Fisher Scientific, #Z25051	1/100	
**IgG controls**	**Reference**	**Dilution**	**Clone**
Mouse IgG1 isotype control FAP	R&D Systems, #MAB002	1/200	11711
Alexa Fluor® 700 Mouse IgG1, κ- Isotype control CD29	BioLegend, #400144	1/25	400144
Alexa Fluor 405 Mouse IgG1 Isotype Control ANTXR1	Novus Biologicals #IC002V	1/25	IC002V
BUV737 Mouse IgG1 Isotype control SDC1	BD Biosciences #612758	1/25	X40
Alexa Fluor 594 Mouse IgG2A Isotype Control GPC3	R&D systems #IC003T	1/20	IC003T
Alexa Fluor 488 Mouse IgG2B Isotype Control DLK1	R&D systems #IC0041G	1/20	IC0041G
BV711 Mouse IgG1 Isotype Control CD9	BD Biosciences #563044	1/100	563044
BV786 Mouse IgG1 Isotype Control CD74	BD Biosciences #563330	1/100	563330
REA control Antibody, human IgG1, PE isotype control LAMP5	Miltenyi Biotec #130-104-613	1/10	REA293
**Antibodies for NK cells characterization**	**Reference**	**Dilution**	**Clone**
APCcy7 Mouse Anti-human CD45	BD Biosciences #557833	1/50	2D1
Alexa Fluor 700 Mouse anti-human CD3	BD Biosciences #557943	1/50	UCHT1
BV510 Mouse anti-human CD14	BD Biosciences #563079	1/50	MφP9
PerCP-Cyanine5.5 Anti-human CD19	BD Biosciences #561295	1/50	HIB19
BV650 Mouse Anti-human CD16	BD Biosciences #563692	1/50	3G8
BUV395 Mouse Anti-human CD56	BD Biosciences #563554	1/50	NCAM16.2
BV786 Mouse Anti-human NKG2A	BD Biosciences #747917	1/50	131411
PE anti-human Granzyme B	BD Biosciences #561142	1/50	GB11
Alexa Fluor 488 Anti human Perforin	BD Biosciences #563764	1/50	δG9
**IgG controls**	**Reference**	**Dilution**	**Clone**
BV650 Mouse IgG1 Isotype Control (CD16)	BD Biosciences #563231	1/50	X40
BUV395 Mouse IgG2b Isotype Control (CD56)	BD Biosciences #563558	1/50	27-35
BV786 Mouse IgG1Isotype Control (NKG2A)	BD Biosciences #563330	1/50	X40
PE Mouse IgG1 Isotype Control (Granzyme B)	BD Biosciences #555749	1/50	MOPC-21
Alexa Fluor 488 Mouse IgG2b Isotype Control (Perforin)	BD Biosciences #558716	1/50	27-35
**Antibodies for macrophages subsets characterization**	**Reference**	**Dilution**	**Clone**
APCcy7 Mouse Anti-human CD45	BD Biosciences #557833	1/50	2D1
Alexa Fluor 700 Mouse anti-human CD3	BD Biosciences #557943	1/50	UCHT1
PEcy7 Mouse anti-human CD14	BD Biosciences #557742	1/50	M5E2
PerCP-Cyanine5.5 Anti-human CD19	BD Biosciences #561295	1/50	HIB19
BV650 Mouse Anti-human CD16	BD Biosciences #563692	1/50	3G8
BUV395 Mouse Anti-human CD56	BD Biosciences #563554	1/50	563554
PE anti-human FOLR2	Biolegend #391704	1/50	94b
Unconjugated anti-human TREM2	Novus Biologicals #MAB17291-100	1/50	237920
APC Goat anti-rat IgG secondary antibody	Novus Biologicals #F0105B	1/50	
**IgG controls**	**Reference**	**Dilution**	**Clone**
BV650 Mouse IgG1 Isotype Control (CD16)	BD Biosciences #563231	1/50	X40
BUV395 Mouse IgG2b Isotype Control (CD56)	BD Biosciences #563558	1/50	27-35
PEcy7 Mouse IgG2a Isotype Control	BD Biosciences #557907	1/50	G155-178
PE Mouse IgG1 Isotype Control	Biolegend #400112	1/100	400112
Unconjugated Rat IgG2b Isotype Control	Novus Biologicals #MAB0061	1/50	
**Antibodies for Regulatory T cells characterization**	**Reference**	**Dilution**	**Clone**
APCcy7 Mouse Anti-human CD45	BD Biosciences #557833	1/50	2D1
Alexa Fluor 700 Mouse anti-human CD3	BD Biosciences #557943	1/50	UCHT1
APC Anti human CD4	Miltenyi #130-113-210	1/10	VIT4
PE anti-human CD25	Miltenyi #130-113-282	1/20	4.00E+03
BUV737 Anti human PD-1	BD Biosciences #612791	1/50	EH12.1
PEcy5 Anti human CTLA4	BD Biosciences #555854	1/50	BNI3
AF488 Anti human FOXP3	Thermofischer Scientific #53-4776-42	1/40	PCH101
**IgG controls**	**Reference**	**Dilution**	**Clone**
PE Isotype Control Antibody, mouse IgG2b	Miltenyi #130-092-215	1/20	IS6-11E5.11
BUV737 Mouse IgG1, κ Isotype Control	BD Biosciences #564299	1/50	X40
PE-Cy5 Mouse IgG2a, κ Isotype Control	BD Biosciences, #555575	1/50	G155-178
Rat IgG2a kappa Isotype Control (eBR2a), Alexa Fluor 488	eBiosciences, #53-4321-80	1/200	eBR2a
**Antibodies for CAF-S1 clusters isolation**	**Reference**	**Dilution**	**Clone**
Alexa Fluor 405 TEM8/ANTXR1 antibody	Novus Biologicals #NB100-56585AF405	1/25	200C1339(SB20)
APC anti-human LAMP-5 antibody	Miltenyi Biotec #130-109-204	1/10	REA590
Alexa Fluor 700 anti-human GPC3 antibody	R&D systems #FAB2119N	1/25	307801
FITC Mouse anti-human CD74	BD Biosciences #555540	1/50	MB741

Supplementary Table 3: List of primary antibodies used in this study

List of primary antibodies used for multicolor flow cytometry and cell sorting in order to characterize FAP+ CAF clusters and immune cell populations. Each antibody was titrated separately to find the best concentration to use. Appropriate isotype controls corresponding to the matched antibody were also used.

Uncropped images for western blot shown in Supplementary Fig. 2L

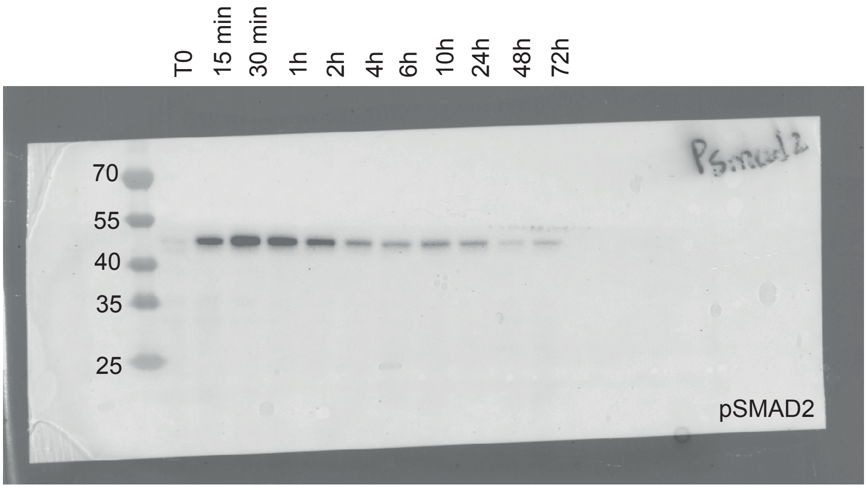

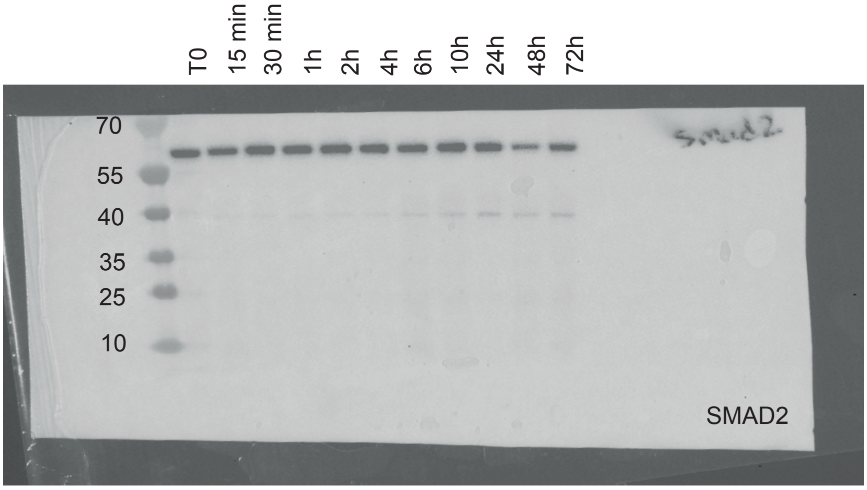

REFERENCES

1. Kieffer Y*, et al.* Single-Cell Analysis Reveals Fibroblast Clusters Linked to Immunotherapy Resistance in Cancer. *Cancer Discov* **10**, 1330-1351 (2020).

2. Bassez A*, et al.* A single-cell map of intratumoral changes during anti-PD1 treatment of patients with breast cancer. *Nat Med* **27**, 820-832 (2021).

3. Wu SZ*, et al.* Stromal cell diversity associated with immune evasion in human triple-negative breast cancer. *EMBO J* **39**, e104063 (2020).

4. Pal B*, et al.* A single-cell RNA expression atlas of normal, preneoplastic and tumorigenic states in the human breast. *EMBO J* **40**, e107333 (2021).

5. Wu SZ*, et al.* A single-cell and spatially resolved atlas of human breast cancers. *Nat Genet* **53**, 1334-1347 (2021).

6. Coutant A*, et al.* Spatial Transcriptomics Reveal Pitfalls and Opportunities for the Detection of Rare High-Plasticity Breast Cancer Subtypes. *Lab Invest* **103**, 100258 (2023).
