# Supplementary material for: Deciphering the spatial landscape and plasticity of immunosuppressive fibroblasts in breast cancer
Source: Nat Commun. 2024 Apr 1;15:2806. doi: 10.1038/s41467-024-47068-z (PMC10984943; doi:10.1038/s41467-024-47068-z)
Supplement: Supplementary file 3 — Reporting Summary [file 41467_2024_47068_MOESM3_ESM.pdf]

nature portfolio

Corresponding author(s): Fatima Mechta-Grigoriou

Last updated by author(s): Feb 23, 2024

Reporting Summary

Nature Portfolio wishes to improve the reproducibility of the work that we publish. This form provides structure for consistency and transparency in reporting. For further information on Nature Portfolio policies, see our Editorial Policies and the Editorial Policy Checklist.

Statistics

For all statistical analyses, confirm that the following items are present in the figure legend, table legend, main text, or Methods section.

n/a	Confirmed	
☐	☒	The exact sample size (*n*) for each experimental group/condition, given as a discrete number and unit of measurement
☐	☒	A statement on whether measurements were taken from distinct samples or whether the same sample was measured repeatedly
☐	☒	The statistical test(s) used AND whether they are one- or two-sided<br>*Only common tests should be described solely by name; describe more complex techniques in the Methods section.*
☐	☒	A description of all covariates tested
☐	☒	A description of any assumptions or corrections, such as tests of normality and adjustment for multiple comparisons
☐	☒	A full description of the statistical parameters including central tendency (e.g. means) or other basic estimates (e.g. regression coefficient) AND variation (e.g. standard deviation) or associated estimates of uncertainty (e.g. confidence intervals)
☐	☒	For null hypothesis testing, the test statistic (e.g. *F*, *t*, *r*) with confidence intervals, effect sizes, degrees of freedom and *P* value noted<br>*Give P values as exact values whenever suitable.*
☐	☒	For Bayesian analysis, information on the choice of priors and Markov chain Monte Carlo settings
☒	☐	For hierarchical and complex designs, identification of the appropriate level for tests and full reporting of outcomes
☐	☒	Estimates of effect sizes (e.g. Cohen's *d*, Pearson's *r*), indicating how they were calculated

Our web collection on statistics for biologists contains articles on many of the points above.

Software and code

Policy information about availability of computer code

Data collection	Flow Cytometry data were acquired using Fortessa LSRII (BD Biosciences)<br>Western Blot images were acquired using Chemidoc (Biorad)<br>ScRNAseq Data were obtained using Chromium system (10X genomics)<br>Spatial transcriptomic data were obtained using visium spatial gene expression assay (10X genomics)
Data analysis	Flow cytometry analysis was performed using Flowjo (V10.10.0)<br>Western Blot images were analyzed using Fiji (2.1.0/1.53c)<br>Quantification graphs were generated using GraphPad (Prism 9.5.1)<br>Single-cell RNA sequencing: Cell Ranger version 2.1.1 (10X Genomics), Seurat version 4.3<br>Spatial transcriptomics: SpaceRanger version 1.2.2 (10X Genomics), Seurat version 4.3<br>Custom code: https://doi.org/10.6084/m9.figshare.25092977

For manuscripts utilizing custom algorithms or software that are central to the research but not yet described in published literature, software must be made available to editors and reviewers. We strongly encourage code deposition in a community repository (e.g. GitHub). See the Nature Portfolio guidelines for submitting code & software for further information.

Data

Policy information about availability of data

All manuscripts must include a data availability statement. This statement should provide the following information, where applicable:

- Accession codes, unique identifiers, or web links for publicly available datasets
- A description of any restrictions on data availability
- For clinical datasets or third party data, please ensure that the statement adheres to our policy

Raw scRNA-seq data from CAP, spatial transcriptomic data, Bulk RNAseq from cultured fibroblasts and the INVADE cohort are available on European Genome-Phenome Archive platform (https://ega-archive.org) under the controlled accession numbers: EGAS50000000220 and EGAS50000000219. The controlled access is required as raw data contain identifying patient information. Data access can be granted via the EGA with completion of an institute data transfer agreement. Processed scRNA-seq data are available from the Figshare data repository link: https://doi.org/10.6084/m9.figshare.20348712. Spatial transcriptomics data generated in this study are available from the Figshare data repository link: https://doi.org/10.6084/m9.figshare.21591429.

Count data from bulk RNA-seq from DCIS, MI-DCIS and IBC (INVADE cohort) are available from the Figshare data repository link: https://doi.org/10.6084/m9.figshare.21591351. Gene expression data and associated clinical data from TBCRC 038 were retrieved from {Strand, 2022 #229}. Processed scRNA-seq datasets from PDAC and BC mice model were recovered from the ArrayExpress repository under the accession number E-MTAB-12036, and from the Gene Expression Omnibus (GEO) accession GSE149636, respectively. Processed scRNA-seq data from {Wu, 2021 #171} were downloaded through the Broad Institute Single Cell portal at https://singlecell.broadinstitute.org/single_cell/study/SCP1039. The scRNA-Seq data from {Wu, 2020 #172} have been recovered from the European Nucleotide Archive under the accession code PRJEB35405. Processed scRNA-seq from {Pal, 2021 #185} were recovered from GEO series GSE161529. Source data are provided with this paper.

Research involving human participants, their data, or biological material

Policy information about studies with human participants or human data. See also policy information about sex, gender (identity/presentation), and sexual orientation and race, ethnicity and racism.

Reporting on sex and gender	Our study exclusively involved female patients as its focus on breast cancer.<br><br>For functionnal assays in vitro, human donor immune cells were obtained from both sexes and sex was not considered in the study design.
Reporting on race, ethnicity, or other socially relevant groupings	we did not use any socially significant categorizations in this study
Population characteristics	Detailed description of retrospective and prospective cohorts of breast cancer patients are available in Supplementary table S1 and S2 and in the Methods Section
Recruitment	Breast cancer patients were included prospectively in our study. No participant selection was applied . we included in our cohorts all patients diagnosed with ductal breast carcinoma for whom available biological samples were available.<br><br>All fresh samples were collected by a referent pathologist. The surgical residues, available after histopathological analyses and used in our manuscript, were not required for diagnosis. There was no interference with clinical practice. All patients treated at Institut Curie were informed orally and through an informative flyer, that their biological samples, collected through standard clinical practice, could be used for research purposes, and that by not opposing this use, they accept it.<br><br>Clinical features of prospective cohorts are listed in Supplementary Table 1 and in Methods section
Ethics oversight	The study developed here is based on samples taken from surgical residues available after histopathologic analyses and not required for diagnosis. There is no interference with clinical practice. Analysis of tumor samples was performed in accordance with the relevant national law and with recognized ethical guidelines (Declaration of Helsinki) on the protection of people taking part in biomedical research. All patients with BC hospitalized at Institut Curie received a welcome booklet explaining that their samples may be used for research purposes. All patients included in our study were thus informed by their referring oncologist that biological samples collected through standard clinical practice could be used for research purposes and they gave their verbal informed consent. In case of patient refusal, which could be either orally expressed or written, residual tumor samples were not included in our study. Human experimental procedures for analyses of tumor microenvironment by F. Mechta-Grigoriou's lab were approved by the Institutional Review Board and Ethics committee of the Institut Curie Hospital group (approval February 12, 2014) and CNIL (Commission Nationale de l'informatique et des Libertés approval no.: 1674356 delivered March 30, 2013). The Biological Resource Centre (BRC) is part of the Pathology Department in the Diagnostic and Theragnostic Medicine Department headed by Dr. A. Vincent-Salomon. BRC is authorized to store and manage human biological samples according to French legislation. The BRC has declared defined sample collections that are continuously incremented as and when patient consent forms are obtained (declaration number: DC-2008-57). The BRC follows all currently required national and international ethical rules, including the Declaration of Helsinki. The BRC has also been accredited with the AFNOR NFS-96-900 quality label (renewed and currently valid until 2021). All samples are pseudo-anonymous when they arrive from the BRC in the lab. In addition, the BRC collections have been declared to the CNIL (approval no. 1487390 delivered February 28, 2011).

Note that full information on the approval of the study protocol must also be provided in the manuscript.

Field-specific reporting

Please select the one below that is the best fit for your research. If you are not sure, read the appropriate sections before making your selection.

- [x] Life sciences
- [ ] Behavioural & social sciences
- [ ] Ecological, evolutionary & environmental sciences

For a reference copy of the document with all sections, see nature.com/documents/nr-reporting-summary-flat.pdf

Life sciences study design

All studies must disclose on these points even when the disclosure is negative.

Sample size

For Flow cytometry analysis on patients samples, the sample size was chosen based on sample availability. These patients were not selected. Statistical analysis were used to define the sample size sufficient to get significant differences
For co-culture functionnal assays in vitro, each experiment was verified at least in three biological replicates.
As primary CAF are short-term cultures, we used different CAF cells isolated from fresh breast cancer samples to generate independent experiments

Data exclusions

No data were excluded from the analyses, except poor quality cells (as explained in the Methods Section of the paper "Building a reference breast cancer atlas / Quality control and processing"

Replication

We generated several biological replicates when it was possible.
For flow cytomery analysis on primary patients samples, we recieved only one piece of tumor per patient which we processed to analyze both the content of CAF clusters and macrophages subsets. therefore, for each patient, we have one unique analysis of this type.
For these experiments , we analyzed n = 87 primary tumors, 25 of which included macrophages subsets content analysis (depending on the size of tuor samples and the number of cells obtained after digestion)

For co-culture functional assays in vitro we analyzed:
co-culture of CAF-S1 clusters with CD14+ monocytes: n = 9 independent experiments, CAF-S1 clusters isolated from 3 patients samples, 3 PBMC donors
co-culture of CAF-S1 clusters with CD4+ CD25+ T cells: n = 8 independent experiments, CAF-S1 clusters isolated from 3 patients samples, 3 PBMC donors => 2 CAF-S1 primary cells were analyzed with three different PBMC donors each, and one CAF-S1 primary cell line was analyzed with two PBMC donor
co-culture of CAF-S1 clusters with NK cells: n = 7 independent experiments, CAF-S1 clusters isolated from 3 patients samples, 3 PBMC donors => 2 CAF-S1 primary cells were analyzed with two different PBMC donors each, and one CAF-S1 primary cell line was analyzed with three PBMC donor
migration: n = 4 independent experiments, CAF-S1 clusters isolated from 3 patients samples, 3 PBMC donors => 1 CAF-S1 primary cells were analyzed with three different PBMC donors each, and two CAF-S1 primary cell line was analyzed with one PBMC donor

For CAF-S1 clusters co-culture with breast cancer cell lines and MCF10A breast epithelial cells: n=3 independent experiments (CAF-S1 clusters isolated from 3 patients samples)

The number of replicates generated for in vitro studies are provided in the respective Figure legends.

Randomization

For all in vitro and coculture studies, all the FAP+ CAF cluster were isolated from the same BC patient and were cocultured in the same time with immune cells isolated from the same healthy PBMC donors. Healthy donors were randomly selected and blind to the investigator.
Etablissement Francais du Sang was in charge to collect and deliver blood from healthy donor to Institut Curie
For patients samples, the analysis depend on the size of tumor samples and the number of cells obtained after processing.

Blinding

As there was no subjective measurements in our experiments, no blinding was performed.

Reporting for specific materials, systems and methods

We require information from authors about some types of materials, experimental systems and methods used in many studies. Here, indicate whether each material, system or method listed is relevant to your study. If you are not sure if a list item applies to your research, read the appropriate section before selecting a response.

Materials & experimental systems

n/a	Involved in the study	
	☒	Antibodies
	☒	Eukaryotic cell lines
☒		Palaeontology and archaeology
☒		Animals and other organisms
	☒	Clinical data
☒		Dual use research of concern
☒		Plants

Methods

n/a	Involved in the study	
☒		ChIP-seq
	☒	Flow cytometry
☒		MRI-based neuroimaging

Antibodies

Antibodies used

All antibodies used in this study are listed in Supplementary Table S3

Antibodies for CAF-S1 clusters characterization Reference Dilution
Brilliant Violet 605 anti-human CD326 (EpCAM) BioLegend, #324224, clone 9C4, Dilution: 1/50
PE/Cyanine7 anti-human CD31 antibody BioLegend, #303118, clone WM59, Dilution:1/100
BUV395 Anti-human CD45 BD Biosciences, #563792 clone HI30, Dilution:1/50
PerCP/Cyanine5.5 anti-human CD235a Biolegend, #349110 clone HI264, Dilution:1/50
Human Fibroblast Activation Proetin Alpha/FAP Antibody R&D Systems, #MAB3715 Clone # 427819 dilution: 1/100
Alexa Fluor700 anti-human CD29 BioLegend, #303020 clone TS2/16 dilution: 1/100
Alexa Fluor 405 TEM8/ANTXR1 antibody Novus Biologicals #NB100-56585AF405 clone 200C1339(SB20) dilution: 1/25
BUV737 anti human CD138 (SDC1) BD Biosciences #612834 clone MI15 dilution: 1/25
Human Glypican 3 Alexa Fluor 594 Antibody R&D systems #FAB2119T , Clone # 307801, dilution: 1/20
Human DLK1 Alex Fluor 488 Antibody R&D systems #FAB1144G Clone # 211309, dilution 1/20
BV711 Anti-human CD9 BD Biosciences #743050, clone M-L13, dilution: 1/100
BV786 Anti-human CD74 BD Biosciences #743736, clone LN2, dilution 1/100
PE anti-human LAMP5 antbody Miltenyi Biotec #130-109-156, clone REA590, dilution 1/10
Fluorescent dye Zenon APC Mouse IgG1 labeling kit Thermo Fisher Scientific, #Z25051 1/100
IgG controls Reference Dilution
Mouse IgG1 isotype control FAP R&D Systems, #MAB002, Clone # 11711, dilution: 1/200
Alexa Fluor® 700 Mouse IgG1, κ- Isotype control CD29 BioLegend, #400144, clone MOPC-21, dilution 1/25
Alexa Fluor 405 Mouse IgG1 Isotype Control ANTXR1 Novus Biologicals #IC002V ,Clone # 11711, dilution 1/25
BUV737 Mouse IgG1 Isotype control SDC1 BD Biosciences #612758, clone X40, dilution 1/25
Alexa Fluor 594 Mouse IgG2A Isotype Control GPC3 R&D systems #IC003T, Clone # 20102 dilution 1/20
Alexa Fluor 488 Mouse IgG2B Isotype Control DLK1 R&D systems #IC0041G; Clone # 133303, dilution 1/20
BV711 Mouse IgG1 Isotype Control CD74 BD Biosciences #563044, clone X40, dilution 1/100
BV786 Mouse IgG1 Isotype Control CD9 BD Biosciences #563330, clone X40, dilution 1/100
REA control Antibody, human IgG1, PE isotype control LAMP5 Miltenyi Biotec #130-104-613, clone REA293, dilution 1/10

Antibodies for NK cells characterization Reference Dilution
APCcy7 Mouse Anti-human CD45 BD Biosciences #557833 , clone 2D1, dilution 1/50
Alexa Fluor 700 Mouse anti-human CD3 BD Biosciences #557943, clone UCHT1, dilution 1/50
BV510 Mouse anti-human CD14 BD Biosciences #563079, clone MϕP9, dilution 1/50
PerCP-Cyanine5.5 Anti-human CD19 BD Biosciences #561295, clone HIB19, dilution 1/50
BV650 Mouse Anti-human CD16 BD Biosciences #563692, clone 3G8, dilution 1/50
BUV395 Mouse Anti-human CD56 BD Biosciences #563554 , clone NCAM16.2, dilution 1/50
BV786 Mouse Anti-human NKG2A BD Biosciences #747917, clone 131411, dilution 1/50
PE anti-human Granzyme B BD Biosciences #561142 , clone GB11, dilution: 1/50
Alexa Fluor 488 Anti human Perforin BD Biosciences #563764, clone δG9, dilution 1/50
IgG controls Reference Dilution
BV650 Mouse IgG1 Isotype Control (CD16) BD Biosciences #563231, clone X40, dilution 1/50
BUV395 Mouse IgG2b Isotype Control (CD56) BD Biosciences #563558 , clone 27-35, dilution 1/50
BV786 Mouse IgG1Isotype Control (NKG2A) BD Biosciences #563330, clone X40, dilution 1/50
PE Mouse IgG1 Isotype Control (Granzyme B) BD Biosciences #555749, clone MOPC-21, dilution 1/50
Alexa Fluor 488 Mouse IgG2b Isotype Control (Perforin) BD Biosciences #558716, clone 27-35, dilution 1/50

Antibodies for macrophages subsets characterization Reference Dilution
APCcy7 Mouse Anti-human CD45 BD Biosciences #557833, clone 2D1, dilution 1/50
Alexa Fluor 700 Mouse anti-human CD3 BD Biosciences #557943, clone UCHT1, dilution 1/50
PEcy7 Mouse anti-human CD14 BD Biosciences #557742, clone M5E2, dilution 1/50
PerCP-Cyanine5.5 Anti-human CD19 BD Biosciences #561295, clone HIB19, dilution 1/50
BV650 Mouse Anti-human CD16 BD Biosciences #563692, clone 3G8, dilution 1/50

BUV395 Mouse Anti-human CD56 BD Biosciences #563554; clone 563554, dilution 1/50
PE anti-human FOLR2 Biolegend #391704, clone 94b, dilution 1/50
Unconjugated anti-human TREM2 Novus Biologicals #MAB17291-100 , Clone # 237920, dilution 1/50
APC Goat anti-rat IgG secondary antibody Novus Biologicals #F0105B 1/50
IgG controls Reference Dilution
BV650 Mouse IgG1 Isotype Control (CD16) BD Biosciences #563231, clone X40, dilution 1/50
BUV395 Mouse IgG2b Isotype Control (CD56) BD Biosciences #563558, clone 27-35, dilution 1/50
PEcy7 Mouse IgG2a Isotype Control BD Biosciences #557907, clone G155-178, dilution 1/50
PE Mouse IgG1 Isotype Control Biolegend #400112, clone 400112, dilution 1/100
Unconjugated Rat IgG2b Isotype Control Novus Biologicals #MAB0061 1/50

Antibodies for Regulatory T cells characterization Reference Dilution
APCcy7 Mouse Anti-human CD45 BD Biosciences #557833, clone 2D1, dilution: 1/50
Alexa Fluor 700 Mouse anti-human CD3 BD Biosciences #557943, clone UCHT1, dilution 1/50
APC Anti human APC Miltenyi #130-113-210, clone VIT4, dilution 1/10
PE anti-human CD25 Miltenyi #130-113-282, clone 4E3 , dilution 1/20
BUV737 Anti human PD-1 BD Biosciences #612791 , clone EH12.1, dilution 1/50
PEcy5 Anti human CTLA4 BD Biosciences #555854, clone BNI3, dilution 1/50
AF488 Anti human FOXP3 Thermofischer Scientific #53-4776-42, clone PCH101, dilution 1/40
IgG controls Reference Dilution
PE Isotype Control Antibody, mouse IgG2b Miltenyi #130-092-215, clone
IS6-11E5.11, dilution 1/20
BUV737 Mouse IgG1, κ Isotype Control BD Biosciences #564299, clone X40, dilution 1/50
PE-Cy5 Mouse IgG2a, κ Isotype Control BD Biosciences, #555575, clone G155-178, dilution 1/50
Rat IgG2a kappa Isotype Control (eBR2a), Alexa Fluor 488 eBiosciences, #53-4321-80, clone eBR2a, dilution 1/200

Antibodies for CAF-S1 clusters isolation Reference Dilution
Alexa Fluor 405 TEM8/ANTXR1 antibody Novus Biologicals #NB100-56585AF405 clone 200C1339(SB20), dilution 1/25
APC anti-human LAMP-5 antibody Miltenyi Biotec #130-109-204, clone REA590, dilution 1/10
Alexa Fluor 700 anti-human GPC3 antibody R&D systems #FAB2119N, Clone # 307801, dilution 1/25
FITC Mouse anti-human CD74 BD Biosciences #555540, clone MB741, dilution 1/50

Antibodies for WB analysis Reference Dilution
Human DPPIV/CD26 Antibody R&D systems #AF1180 1/1000
Human YAP (D8H1X) Rabbit monoclonal antibody Cell Signaling #14074 1/1000
SMAD2 (D43B4) XP Rabbit monoclonal antibody Cell signaling #5339 1/1000
Phospho-SMAD2 (Ser465/467) Rabbit monoclonal antibody Cell signaling #3108 1/1000
Monoclonal antibody anti human β-actin Sigma #A5441 1/10000
TGF-β Receptor II Rabbit monoclonal antibody Cell signaling #41896 1/1000

Validation

For commercially available antibodies, validation has been performed by the manufacturer and corresponding certificates of analysis are listed below

Antibodies for CAF-S1 clusters characterization Reference Dilution
Brilliant Violet 605 anti-human CD326 (EpCAM) BioLegend, #324224 1/50 https://d1spbj2x7qk4bg.cloudfront.net/fr-fr/search-results/brilliant-violet-605-anti-human-cd326-epcam-antibody-8886?pdf=true&displayInline=true&leftRightMargin=15&topBottomMargin=15&filename=Brilliant%20Violet%20605™%20anti-human%20CD326%20(EpCAM)%20Antibody.pdf&v=20230803063053
PE/Cyanine7 anti-human CD31 antibody BioLegend, #303118 1/100 https://d1spbj2x7qk4bg.cloudfront.net/fr-fr/products/pe-cyanine7-anti-human-cd31-antibody-6124?pdf=true&displayInline=true&leftRightMargin=15&topBottomMargin=15&filename=PE/Cyanine7%20anti-human%20CD31%20Antibody.pdf&v=20230114013553
BUV395 Anti-human CD45 BD Biosciences, #563792 1/50 https://www.bdbiosciences.com/content/bdb/paths/generate-tds-document.fr.563792.pdf
PerCP/Cyanine5.5 anti-human CD235a Biolegend, #349110 1/50 https://d1spbj2x7qk4bg.cloudfront.net/fr-fr/products/percp-cyanine5-5-anti-human-cd235a-glycophorin-a-antibody-9002?pdf=true&displayInline=true&leftRightMargin=15&topBottomMargin=15&filename=PerCP/Cyanine5.5%20anti-human%20CD235a%20(Glycophorin%20A)%20Antibody.pdf&v=20231027063032
Human Fibroblast Activation Proetin Alpha/FAP Antibody R&D Systems, #MAB3715 1/100 https://resources.rndsystems.com/pdfs/datasheets/mab3715.pdf?v=20231228&_ga=2.205219929.838383242.1703755343-649884436.1703755343
Alexa Fluor700 anti-human CD29 BioLegend, #303020 1/100 https://d1spbj2x7qk4bg.cloudfront.net/fr-fr/products/alexa-fluor-700-anti-human-cd29-antibody-3420?pdf=true&displayInline=true&leftRightMargin=15&topBottomMargin=15&filename=Alexa%20Fluor®%20700%20anti-human%20CD29%20Antibody.pdf&v=20231223073221
Alexa Fluor 405 TEM8/ANTXR1 antibody Novus Biologicals #NB100-56585AF405 1/25 https://www.novusbio.com/PDFs2/NB100-56585AF405.pdf?_ga=2.200941927.838383242.1703755343-649884436.1703755343
BUV737 anti human CD138 (SDC1) BD Biosciences #612834 1/25 https://www.bdbiosciences.com/content/bdb/paths/generate-tds-document.fr.612834.pdf
Human Glypican 3 Alexa Fluor 594 Antibody R&D systems #FAB2119T 1/20 https://resources.rndsystems.com/pdfs/datasheets/fab2119t.pdf?v=20231228&_ga=2.200941927.838383242.1703755343-649884436.1703755343
Human DLK1 Alex Fluor 488 Antibody R&D systems #FAB1144G 1/20 https://resources.rndsystems.com/pdfs/datasheets/fab1144g.pdf?v=20231228&_ga=2.126714308.838383242.1703755343-649884436.1703755343

BV711 Anti-human CD9 BD Biosciences #743050 1/100 https://www.bdbiosciences.com/en-fr/products/reagents/flow-cytometry-reagents/research-reagents/single-color-antibodies-ruo/bv711-mouse-anti-human-cd9.743050
BV786 Anti-human CD74 BD Biosciences #743736 1/100 https://www.bdbiosciences.com/content/bdb/paths/generate-tds-document.fr.743736.pdf
PE anti-human LAMP5 antbody Miltenyi Biotec #130-109-156 1/10 https://static.miltenyibiotec.com/asset/150655405641/document_db4q4t973l3h9f7prquf2cga1t?content-disposition=inline
Fluorescent dye Zenon APC Mouse IgG1 labeling kit Thermo Fisher Scientific, #Z25051 1/100 https://www.thermofisher.com/document-connect/document-connect.html?url=https://assets.thermofisher.com/TFS-Assets%2FLSG%2Fmanuals%2FMAN0025408_ZenonMouseIgGLabelingKits_UG.pdf
IgG controls Reference Dilution
Mouse IgG1 isotype control FAP R&D Systems, #MAB002 1/200 https://resources.rndsystems.com/pdfs/datasheets/mab002.pdf?v=20231228&_ga=2.130187846.838383242.1703755343-649884436.1703755343
Alexa Fluor® 700 Mouse IgG1, κ- Isotype control CD29 BioLegend, #400144 1/25 https://d1spbj2x7qk4bg.cloudfront.net/fr-fr/products/alexa-fluor-700-mouse-igg1-kappa-isotype-ctrl-3376?pdf=true&displayInline=true&leftRightMargin=15&topBottomMargin=15&filename=Alexa%20Fluor®%20700%20Mouse%20IgG1,%20κ%20Isotype%20Ctrl%20Antibody.pdf&v=20230714033116
Alexa Fluor 405 Mouse IgG1 Isotype Control ANTXR1 Novus Biologicals #IC002V 1/25 https://resources.rndsystems.com/pdfs/datasheets/ic002v.pdf?_ga=2.130187846.838383242.1703755343-649884436.1703755343
BUV737 Mouse IgG1 Isotype control SDC1 BD Biosciences #612758 1/25
Alexa Fluor 594 Mouse IgG2A Isotype Control GPC3 R&D systems #IC003T 1/20 https://resources.rndsystems.com/pdfs/datasheets/ic003t.pdf?v=20231228&_ga=2.95563477.838383242.1703755343-649884436.1703755343
Alexa Fluor 488 Mouse IgG2B Isotype Control DLK1 R&D systems #IC0041G 1/20 https://resources.rndsystems.com/pdfs/datasheets/ic0041g.pdf?v=20231228&_ga=2.122389186.838383242.1703755343-649884436.1703755343
BV711 Mouse IgG1 Isotype Control CD74 BD Biosciences #563044 1/100 https://www.bdbiosciences.com/content/bdb/paths/generate-tds-document.fr.563044.pdf
BV786 Mouse IgG1 Isotype Control CD9 BD Biosciences #563330 1/100 https://www.bdbiosciences.com/content/bdb/paths/generate-tds-document.fr.563330.pdf
REA control Antibody, human IgG1, PE isotype control LAMP5 Miltenyi Biotec #130-104-613 1/10 https://static.miltenyibiotec.com/asset/150655405641/document_otcau3sihp12r1r4gdipslqk63?content-disposition=inline

Antibodies for NK cells characterization Reference Dilution
APCcy7 Mouse Anti-human CD45 BD Biosciences #557833 1/50 https://www.bdbiosciences.com/content/bdb/paths/generate-tds-document.fr.557833.pdf
Alexa Fluor 700 Mouse anti-human CD3 BD Biosciences #557943 1/50 https://www.bdbiosciences.com/content/dam/bdb/products/global/reagents/flow-cytometry-reagents/research-reagents/single-color-antibodies-ruo/557xxx/5579xx/557943_base/pdf/557943.pdf
BV510 Mouse anti-human CD14 BD Biosciences #563079 1/50 https://www.bdbiosciences.com/content/dam/bdb/products/global/reagents/flow-cytometry-reagents/research-reagents/single-color-antibodies-ruo/563xxx/5630xx/563079_base/pdf/563079.pdf
PerCP-Cyanine5.5 Anti-human CD19 BD Biosciences #561295 1/50 https://www.bdbiosciences.com/content/dam/bdb/products/global/reagents/flow-cytometry-reagents/research-reagents/single-color-antibodies-ruo/561xxx/5612xx/561295_base/pdf/561295.pdf
BV650 Mouse Anti-human CD16 BD Biosciences #563692 1/50 https://www.bdbiosciences.com/content/dam/bdb/products/global/reagents/flow-cytometry-reagents/research-reagents/single-color-antibodies-ruo/563xxx/5636xx/563691_base/pdf/563692.pdf
BUV395 Mouse Anti-human CD56 BD Biosciences #563554 1/50 https://www.bdbiosciences.com/content/dam/bdb/products/global/reagents/flow-cytometry-reagents/research-reagents/single-color-antibodies-ruo/563xxx/5635xx/563554_base/pdf/563554.pdf
BV786 Mouse Anti-human NKG2A BD Biosciences #747917 1/50 https://www.bdbiosciences.com/content/bdb/paths/generate-tds-document.fr.747917.pdf
PE anti-human Granzyme B BD Biosciences #561142 1/50 https://www.bdbiosciences.com/content/dam/bdb/products/global/reagents/flow-cytometry-reagents/research-reagents/single-color-antibodies-ruo/561xxx/5611xx/561142_base/pdf/561142.pdf
Alexa Fluor 488 Anti human Perforin BD Biosciences #563764 1/50 https://www.bdbiosciences.com/content/dam/bdb/products/global/reagents/flow-cytometry-reagents/research-reagents/single-color-antibodies-ruo/563xxx/5637xx/563764_base/pdf/563764.pdf
IgG controls Reference Dilution
BV650 Mouse IgG1 Isotype Control (CD16) BD Biosciences #563231 1/50 https://www.bdbiosciences.com/content/dam/bdb/products/global/reagents/flow-cytometry-reagents/research-reagents/flow-cytometry-controls-and-lysates/563xxx/5632xx/563231_base/pdf/563231.pdf
BUV395 Mouse IgG2b Isotype Control (CD56) BD Biosciences #563558 1/50 https://www.bdbiosciences.com/content/dam/bdb/products/global/reagents/flow-cytometry-reagents/research-reagents/flow-cytometry-controls-and-lysates/563xxx/5635xx/563558_base/pdf/563558.pdf
BV786 Mouse IgG1Isotype Control (NKG2A) BD Biosciences #563330 1/50 https://www.bdbiosciences.com/content/dam/bdb/products/global/reagents/flow-cytometry-reagents/research-reagents/flow-cytometry-controls-and-lysates/563xxx/5633xx/563330_base/pdf/563330.pdf
PE Mouse IgG1 Isotype Control (Granzyme B) BD Biosciences #555749 1/50 https://www.bdbiosciences.com/content/dam/bdb/products/global/reagents/flow-cytometry-reagents/research-reagents/flow-cytometry-controls-and-lysates/555xxx/5557xx/555749_base/pdf/555749.pdf
Alexa Fluor 488 Mouse IgG2b Isotype Control (Perforin) BD Biosciences #558716 1/50 https://www.bdbiosciences.com/content/dam/bdb/products/global/reagents/flow-cytometry-reagents/research-reagents/single-color-antibodies-ruo/558xxx/5587xx/558716_base/pdf/558716.pdf

Antibodies for macrophages subsets characterization Reference Dilution
APCcy7 Mouse Anti-human CD45 BD Biosciences #557833 1/50 https://www.bdbiosciences.com/content/dam/bdb/products/global/

reagents/flow-cytometry-reagents/research-reagents/single-color-antibodies-ruo/557xxx/5578xx/557833_base/pdf/557833.pdf
Alexa Fluor 700 Mouse anti-human CD3 BD Biosciences #557943 1/50 https://www.bdbiosciences.com/content/dam/bdb/products/global/reagents/flow-cytometry-reagents/research-reagents/single-color-antibodies-ruo/557xxx/5579xx/557943_base/pdf/557943.pdf
PEcy7 Mouse anti-human CD14 BD Biosciences #557742 1/50 https://www.bdbiosciences.com/content/dam/bdb/products/global/reagents/flow-cytometry-reagents/research-reagents/single-color-antibodies-ruo/557xxx/5577xx/557742_base/pdf/557742.pdf
PerCP-Cyanine5.5 Anti-human CD19 BD Biosciences #561295 1/50 https://www.bdbiosciences.com/content/dam/bdb/products/global/reagents/flow-cytometry-reagents/research-reagents/single-color-antibodies-ruo/561xxx/5612xx/561295_base/pdf/561295.pdf
BV650 Mouse Anti-human CD16 BD Biosciences #563692 1/50 https://www.bdbiosciences.com/content/dam/bdb/products/global/reagents/flow-cytometry-reagents/research-reagents/single-color-antibodies-ruo/563xxx/5636xx/563691_base/pdf/563692.pdf
BUV395 Mouse Anti-human CD56 BD Biosciences #563554 1/50 https://www.bdbiosciences.com/content/dam/bdb/products/global/reagents/flow-cytometry-reagents/research-reagents/single-color-antibodies-ruo/563xxx/5635xx/563554_base/pdf/563554.pdf
PE anti-human FOLR2 Biolegend #391704 1/50 https://d1spbj2x7qk4bg.cloudfront.net/fr-fr/products/pe-anti-human-folate-receptor-beta-fr-beta-antibody-15115?pdf=true&displayInline=true&leftRightMargin=15&topBottomMargin=15&filename=PE%20anti-human%20Folate%20Receptor%20β%20(FR-β)%20Antibody.pdf&v=20230817033058
Unconjugated anti-human TREM2 Novus Biologicals #MAB17291-100 1/50 https://resources.rndsystems.com/pdfs/datasheets/mab17291.pdf?_ga=2.58492643.838383242.1703755343-649884436.1703755343
APC Goat anti-rat IgG secondary antibody Novus Biologicals #F0105B 1/50 https://resources.rndsystems.com/pdfs/datasheets/f0105b.pdf?_ga=2.196879845.838383242.1703755343-649884436.1703755343
IgG controls Reference Dilution
BV650 Mouse IgG1 Isotype Control (CD16) BD Biosciences #563231 1/50 https://www.bdbiosciences.com/content/bdb/paths/generate-tds-document.fr.563231.pdf
BUV395 Mouse IgG2b Isotype Control (CD56) BD Biosciences #563558 1/50 https://www.bdbiosciences.com/content/dam/bdb/products/global/reagents/flow-cytometry-reagents/research-reagents/flow-cytometry-controls-and-lysates/563xxx/5635xx/563558_base/pdf/563558.pdf
PEcy7 Mouse IgG2a Isotype Control BD Biosciences #557907 1/50 https://www.bdbiosciences.com/content/dam/bdb/products/global/reagents/flow-cytometry-reagents/research-reagents/flow-cytometry-controls-and-lysates/557xxx/5579xx/557907_base/pdf/557907.pdf
PE Mouse IgG1 Isotype Control Biolegend #400112 1/100 https://d1spbj2x7qk4bg.cloudfront.net/fr-fr/products/pe-mouse-igg1-kappa-isotype-ctrl-1408?pdf=true&displayInline=true&leftRightMargin=15&topBottomMargin=15&filename=PE%20Mouse%20IgG1,%20κ%20Isotype%20Ctrl%20Antibody.pdf&v=20230714033116
Unconjugated Rat IgG2b Isotype Control Novus Biologicals #MAB0061 1/50 https://resources.rndsystems.com/pdfs/datasheets/mab0061.pdf?_ga=2.196879845.838383242.1703755343-649884436.1703755343

Antibodies for Regulatory T cells characterization Reference Dilution
APCcy7 Mouse Anti-human CD45 BD Biosciences #557833 1/50 https://www.bdbiosciences.com/content/dam/bdb/products/global/reagents/flow-cytometry-reagents/research-reagents/flow-cytometry-controls-and-lysates/563xxx/5632xx/563231_base/pdf/563231.pdf
Alexa Fluor 700 Mouse anti-human CD3 BD Biosciences #557943 1/50 https://www.bdbiosciences.com/content/dam/bdb/products/global/reagents/flow-cytometry-reagents/research-reagents/single-color-antibodies-ruo/557xxx/5579xx/557943_base/pdf/557943.pdf
APC Anti human APC Miltenyi #130-113-210 1/10 https://static.miltenyibiotec.com/asset/150655405641/document_tl49t5csg50k72on4aqdcjfg6a?content-disposition=inline
PE anti-human CD25 Miltenyi #130-113-282 1/20 https://static.miltenyibiotec.com/asset/150655405641/document_mkc8pnoni13oj2ofbdbs8u1g0t?content-disposition=inline
BUV737 Anti human PD-1 BD Biosciences #612791 1/50 https://www.bdbiosciences.com/content/dam/bdb/products/global/reagents/flow-cytometry-reagents/research-reagents/single-color-antibodies-ruo/612xxx/6127xx/612791_base/pdf/612791.pdf
PEcy5 Anti human CTLA4 BD Biosciences #555854 1/50 https://www.bdbiosciences.com/content/dam/bdb/products/global/reagents/flow-cytometry-reagents/research-reagents/single-color-antibodies-ruo/555xxx/5558xx/555854_base/pdf/555854.pdf
AF488 Anti human FOXP3 Thermofischer Scientific #53-4776-42 1/40 https://www.thermofisher.com/order/genome-database/dataSheetPdf?producttype=antibody&productsubtype=antibody_primary&productId=53-4776-42&version=364
IgG controls Reference Dilution
PE Isotype Control Antibody, mouse IgG2b Miltenyi #130-092-215 1/20 https://static.miltenyibiotec.com/asset/150655405641/document_47lo81ihdl3pn4o9ph11ni7m65?content-disposition=inline
BUV737 Mouse IgG1, κ Isotype Control BD Biosciences #564299 1/50 https://www.bdbiosciences.com/en-fr/products/reagents/flow-cytometry-reagents/research-reagents/flow-cytometry-controls-and-lysates/buv737-mouse-igg1-isotype-control.612758
PE-Cy5 Mouse IgG2a, κ Isotype Control BD Biosciences, #555575 1/50 https://www.bdbiosciences.com/content/bdb/paths/generate-tds-document.fr.555575.pdf
Rat IgG2a kappa Isotype Control (eBR2a), Alexa Fluor 488 eBiosciences, #53-4321-80 1/200 https://www.thermofisher.com/order/genome-database/dataSheetPdf?producttype=antibody&productsubtype=antibody_control&productId=53-4321-80&version=364

Antibodies for CAF-S1 clusters isolation Reference Dilution
Alexa Fluor 405 TEM8/ANTXR1 antibody Novus Biologicals #NB100-56585AF405 1/25 https://www.novusbio.com/PDFs2/NB100-56585AF405.pdf?_ga=2.200941927.838383242.1703755343-649884436.1703755343
APC anti-human LAMP-5 antibody Miltenyi Biotec #130-109-204 1/10 https://static.miltenyibiotec.com/asset/150655405641/document_cmjm6fj2kl5tre2kh2iigkko1s?content-disposition=inline
Alexa Fluor 700 anti-human GPC3 antibody R&D systems #FAB2119N 1/25 https://resources.rndsystems.com/pdfs/datasheets/fab2119n.pdf?v=20231228&_ga=2.163417845.838383242.1703755343-649884436.1703755343
FITC Mouse anti-human CD74 BD Biosciences #555540 1/50 https://www.bdbiosciences.com/content/dam/bdb/products/global/reagents/flow-cytometry-reagents/research-reagents/single-color-antibodies-ruo/555xxx/5555xx/555540_base/pdf/555540.pdf

Antibodies for WB analysis Reference Dilution
Human DPPIV/CD26 Antibody R&D systems #AF1180 1/1000 https://resources.rndsystems.com/pdfs/datasheets/af1180.pdf?v=20231228&_ga=2.163417845.838383242.1703755343-649884436.1703755343
Human YAP (D8H1X) Rabbit monoclonal antibody Cell Signaling #14074 1/1000 https://www.cellsignal.com/datasheet.jsp?productId=14074&images=1&size=A4
SMAD2 (D43B4) XP Rabbit monoclonal antibody Cell signaling #5339 1/1000 https://www.cellsignal.com/datasheet.jsp?productId=5339&images=1&size=A4
Phospho-SMAD2 (Ser465/467) Rabbit monoclonal antibody Cell signaling #3108 1/1000 https://www.cellsignal.com/datasheet.jsp?productId=3108&images=1&size=A4
Monoclonal antibody anti human β-actin Sigma #A5441 1/10000 https://www.sigmaaldrich.com/deepweb/assets/sigmaaldrich/product/documents/296/386/a5441dat.pdf
TGF-β Receptor II Rabbit monoclonal antibody Cell signaling #41896 1/1000 https://www.cellsignal.com/datasheet.jsp?productId=41896&images=1&size=A4

Eukaryotic cell lines

Policy information about cell lines and Sex and Gender in Research

Cell line source(s)

Primary CAF-S1 clusters were isolated from breast cancer fresh tumor samples.
Fresh BC samples received after surgery were cut into fragments of approximately 1 mm3, put either in plastic petri dishes or in petri dishes coated with type I collagen at a final concentration of 9 µg/ml (Institut De Biotechnologie Jacques Boy, #207050357) and cultured in DMEM (Gibco, #41966-029) supplemented with 10% heat inactivated FBS (Biosera, #FB-1003-500) and 1% streptomycin and penicillin (Sigma, #p4333) for 2-3 weeks at 37°C. Media was renewed every 3 days during an expansion phase of 2-3 weeks. When fibroblasts reached at least 50% of confluency, they were detached using TrypLE (Gibco, #12605-010), centrifuged at 1200 rpm for 5 min and plated in new plastic plates or collagen-coated plates using DMEM supplemented as above. To separate the different FAP+ CAF clusters, cells in both conditions were collected separately and sorted by BDFACS ARIA III using FAP+ CAF cluster-specific surface markers described in {Kieffer, 2020 #61}. For cell sorting strategy, FAP+ CAF cultured on plastic plates were separated based on ANTXR1 and LAMP5 in 2 distinct clusters, ECM-myCAF (CD29+ FAP+ ANTXR1+ LAMP5-) and TGFb-myCAF (CD29+ FAP+ ANTXR1+ LAMP5+). Cells were stained with an antibody mix containing anti-CD29-Alexa Fluor 700 (1:100, BioLegend, #303020), anti-FAP-APC (1:100, R&D Systems, #MAB3715), anti-ANTXR1-AF405 (1:25, Novus Bio, #NB100-56585AF405) and anti-LAMP5-APC (1:10, Miltenyi Biotec, #130-109-204). We applied similar strategy for cells cultured on collagen-coated plates. FAP+ CAF were sorted in 3 distinct iCAF clusters based on ANTXR1, GPC3 and CD74, defined as followed: Detox-iCAF (CD29+ FAP+ ANTXR1- GPC3+), IL-iCAF (CD29+ FAP+ ANTXR1- GPC3-) and IFN-iCAF (CD29+ FAP+ ANTXR1- GPC3- CD74+). To do so, cells were stained with an antibody mix containing anti-CD29-Alexa Fluor 700 (1:100, BioLegend, #303020), anti-FAP-APC (1:100, R&D Systems, #MAB3715), anti-ANTXR1-AF405 (1:25, Novus Bio, #NB100-56585AF405), anti-GPC3-AF700 (1:25, R&D systems, #FAB2119N) and anti-CD74-FITC (1:50, BD Biosciences, #555540). After sorting, cells were expanded in culture at 37°C in DMEM media supplemented as above, in a humidified 1.5% O2 and 5% CO2 incubator, either on plastic dishes for ECM-myCAF and TGFb-myCAF or on collagen-coated dishes for Detox-iCAF, IL-iCAF and IFNg-CAF. To avoid any change in FAP+ CAF cluster identity, fibroblasts were maintained in the same culture condition after sorting. All experiments using FAP+ CAF primary cell lines were not performed beyond passage 10 to avoid fibroblast senescence.

MCF7, MDA-MB231, T47D and MCF10A breast cell lines were purshased from ATCC.
MCF7 and MDA-MB-231 were cultured in DMEM supplemented with 10% FBS and 1% PS, in a humidified 20% O2 and 5% CO2 incubator
T47D were cultured in RPMI supplemented with 10% FBS and 1% PS, in a humidified 20% O2 and 5% CO2 incubator
MCF10A were cultured in DMEM/F12 media supplemented with 10% FBS and 1% PS, in a humidified 20% O2 and 5% CO2 incubator

Authentication

Identity of primary CAF-S1 fibroblasts in culture was next validated by flow cytometry and bulk-RNAseq

For MCF7, MDA-MB-231 and T47D breast cancer cell lines and for the MCF10A non tumoral epithelial breast cell line, , the cell identity was verified by using the Short Tandem Repeat (STR) DNA profiling (Promega # B9510) method.

Mycoplasma contamination

Cell lines were routinely tested for mycoplasma contamination by mycoplasma detection kit and confirmed that they were negative.

Commonly misidentified lines
(See ICLAC register)

No commonly misidentified cell lines were used.

Clinical data

Policy information about clinical studies

All manuscripts should comply with the ICMJE guidelines for publication of clinical research and a completed CONSORT checklist must be included with all submissions.

Clinical trial registration	Although this study includes clinical data, it is not a clinical trial
Study protocol	N/A
Data collection	The INVADE cohort is a retrospective series of 55 patients, who have been treated at Institut Curie between 1992 and 2014, and underwent surgery for a breast carcinoma prior to any treatment<br>All patients for prospective cohorts 1 and 2 were collected from 2020-2023
Outcomes	N/A

Flow Cytometry

Plots

Confirm that:

- [x] The axis labels state the marker and fluorochrome used (e.g. CD4-FITC).
- [x] The axis scales are clearly visible. Include numbers along axes only for bottom left plot of group (a 'group' is an analysis of identical markers).
- [x] All plots are contour plots with outliers or pseudocolor plots.
- [x] A numerical value for number of cells or percentage (with statistics) is provided.

Methodology

Sample preparation	Fresh human BC samples were collected directly after macroscopic examination and selection of areas of interest by a pathologist. Tumor samples were stored in CO2-independent medium and transferred to the research institute. All tumor samples were processed without any previous knowledge about CAF and immune cell infiltration. Samples were cut into small pieces (around 1mm3) and digested in CO2-independent medium (Gibco #18045-054) supplemented with 5% human serum (BioWest #54190-100), 2 mg/ml of collagenase I (Sigma #C0130), 2 mg/ml of hyaluronidase (Sigma #H3506) and 25 mg/ml of Dnase I (Roche #11284932001) during 45 min at 37 °C with permanent shaking (500 rpm). Cells were then filtrated through a 40 µm cell strainer (Fisher Scientific #223635447) and resuspended in PBS+ solution (PBS, Gibco #14190; EDTA 2 mM, Gibco #15575; Human Serum 1%, BioWest #S4190-100). After centrifugation, cells were counted using BeckmanCell Counter and resuspended to a concentration of 5 x 105 to 1 x106. Cells were first incubated with Live/Dead dye (1:1000, BD Horizon™ Fixable Viability Stain 780 dye, BD Biosciences, #565388 for FAP+ CAF clusters and Fixable Violet Dead Cell Stain Kit, Thermo Fischer, #L34955 for TAM subsets) for 10 min at room temperature (RT) to exclude non-viable cells. After a rapid washing with PBS+, cell suspension was then stained for 20 min at RT with an antibody mix specific pour each cell type. the antibody mix used for each cell type characterization is detailed in the Methods section and the supplementary table S3
Instrument	Fortessa flow cytometry (BD FACSCantoII, Becton Dickinson,USA) was used to acquire Flow cytometry results
Software	Flow cytometry data were analyzed with Flowjo software (FlowJo version 10.4.2 (LLC))
Cell population abundance	At least 5×105 events were recorded.<br>Percentage of CAF are calculated among total viable cells. Percentage of ANTXR1+ cells is calculated among the CAF-S1 population (FAP+ CD29+).<br>Percentage of macrophages subsets is calculated among total CD45+ CD14+ cells.<br>Percentages of NK subsets was calculated among total CD45+ cells<br>Percentage od FOXP3+ regulatory T cells was calculated among total CD3+ T cells
Gating strategy	For flow cytometry analysis of tumor samples, cells were first gated based on their size (FSC-A) and granularity (SSC-A). Cell types were then analyzed on the Live/Dead negative fraction and defined as epithelial (EPCAM+), hematopoietic (CD45+), endothelial (CD31+) and red blood cells (CD235a+). Specific surface markers are then added to the antibody mix to characterize FAP+ CAF clusters and macrophages subsets.<br>FAP+ CAF are identified as CD45- EPCAM- CD31- CD235a- FAP+ CD29+ cells. Among FAP+ CD29+, fibroblasts were separated in two subsets according to ANTXR1 surface marker, inflammatory iCAF (ANTXR1-) and myofibroblastic myCAF (ANTXR1+). Among ANTXR1+ CAF, 3 distinct FAP+ CAF clusters were identified as followed, ECM-myCAF (ANTXR1+ SDC1+ LAMP5-), TGF-myCAF (ANTXR1+ SDC1+/- LAMP5+) and Wound-myCAF (ANTXR1+ SDC1- LAMP5- CD9+). ANTXR1- FAP+ CAF were separated based on DLK1, GPC3 and CD74 protein levels to distinguish Detox-iCAF as DLK1+ GPC3+, IL-iCAF as DLK1+ GPC3- and IFN-iCAF as DLK1- GPC3- CD74+.<br><br>Among total CD45+ hematopoietic cells, CD3, CD19 and CD56 markers were used to exclude T lymphocytes (CD3+), B lymphocytes (CD19+) and NK cells (CD56+). TAM subsets were next characterized as CD14+ CD16+ cells and the percentage of TREM2+ and FOLR2+ macrophages was then evaluated.<br><br>For NK characterization, cells were separated in two subsets according to CD56 and CD16 and defined as cytotoxic NK

(CD16high CD56Med) and noncytotoxic NK (CD16- CD56high)

- [x] Tick this box to confirm that a figure exemplifying the gating strategy is provided in the Supplementary Information.
